# Supplementary material for: Antibacterial Thiopeptide GE2270-Congeners from Nonomuraea jiangxiensis
Source: Molecules. 2022 Dec 23;28(1):101. doi: 10.3390/molecules28010101 (PMC9822014; doi:10.3390/molecules28010101)
Supplement: Supplementary file 1 [file molecules-28-00101-s001.zip › molecules-2036940-supplementary.pdf]

Supplementary information for

Antibacterial Thiopeptide GE2270-Congeners from *Nonomuraea jiangxiensis*

Kuan-Chieh Ching †, Elaine J. Chin †, Mario Wibowo, Zann Y. Tan, Lay-Kien Yang, Deborah C. Seow, Chung-Yan Leong, Veronica W. Ng, Siew-Bee Ng * and Yoganathan Kanagasundaram *

Singapore Institute of Food and Biotechnology Innovation (SIFBI), Agency for Science, Technology and Research (A*STAR), Singapore 138673, Singapore

* Correspondence: ngsb@sifbi.a-star.edu.sg (S.-B.N.); yoganathank@sifbi.a-star.edu.sg (Y.K.); Tel.: +65-869596 (S.-B.N.); +65-869508 (Y.K.)

† These authors contributed equally to this work.

Table S1. NMR spectral data of **3** and **4**.

Table S2. ^{1}H and ^{13}C NMR data of **5** and **6.**

Table S3. ^{1}H and ^{13}C NMR data of **7** and ^{1}H NMR data of **8** and **9**.

Figure S1. UV spectra for compounds **1**–**9**.

Figure S2. (+)-HRESIMS spectra for compounds **1**–**9**.

Figure S3. A. Extracted ion chromatogram (*m/z* 1290.2653 ± 5 ppm), **B.** (+)-HRESIMS spectrum, and **C**. MS/MS spectrum of GE2270A in the extract of *Nonomuraea jiangxiensis* strain A7611.

Figure S4. Selected COSY and HMBC correlations of **3** and **4**.

Figure S5. Selected COSY and HMBC correlations of **5** and **6**.

Figure S6. Selected COSY and HMBC correlations of **7**.

Figure S7. ^{1}H NMR spectrum (DMSO-d_6, 400 MHz) of **1**.

Figure S8. ^{13}C NMR spectrum (DMSO-d_6, 100 MHz) of **1**.

Figure S9. COSY spectrum of **1**.

Figure S10. HSQC spectrum of **1**.

Figure S11. HMBC spectrum of **1**.

Figure S12. ^{1}H NMR spectrum (DMSO-d_6, 400 MHz) of **2**.

Figure S13. ^{13}C NMR spectrum (DMSO-d_6, 100 MHz) of **2**.

Figure S14. COSY spectrum of **2**.

Figure S15. HSQC spectrum of **2**.

Figure S16. HMBC spectrum of **2**.

Figure S17. ^{1}H NMR spectrum (DMSO-d_6, 400 MHz) of **3**.

Figure S18. COSY spectrum of **3**.

Figure S19. HSQC spectrum of **3**.

Figure S20. HMBC spectrum of **3**.

Figure S21. ^{1}H NMR spectrum (DMSO-d_6, 400 MHz) of **4**.

Figure S22. ^{13}C NMR spectrum (DMSO-d_6, 100 MHz) of **4**.

Figure S23. COSY spectrum of **4**.

Figure S24. HSQC spectrum of **4**.

Figure S25. HMBC spectrum of **4**.

Figure S26. ^{1}H NMR spectrum (DMSO-d_6, 400 MHz) of **5**.

Figure S27. ^{13}C NMR spectrum (DMSO-d_6, 100 MHz) of **5**.

Figure S28. COSY spectrum of **5**.

Figure S29. HSQC spectrum of **5**.

Figure S30. HMBC spectrum of **5**.

Figure S31. ^{1}H NMR spectrum (DMSO-d_6, 400 MHz) of **6**.

Figure S32. ^{13}C NMR spectrum (DMSO-d_6, 100 MHz) of **6**.

Figure S33. COSY spectrum of **6**.

Figure S34. HSQC spectrum of **6**.

Figure S35. HMBC spectrum of **6**.

Figure S36. ^{1}H NMR spectrum (DMSO-d_6, 400 MHz) of **7**.

Figure S37. ^{13}C NMR spectrum (DMSO-d_6, 100 MHz) of **7**.

Figure S38. COSY spectrum of **7**.

Figure S39. HSQC spectrum of **7**.

Figure S40. HMBC spectrum of **7**.

Figure S41. ^{1}H NMR spectrum (DMSO-d_6, 400 MHz) of **8**.

Figure S42. ^{1}H NMR spectrum (DMSO-d_6, 400 MHz) of **9**.

Figure S43. Dose response curve of compounds **1-9** against *Klebsiella aerogenes* (ATCC® 13048™), *Pseudomonas aeruginosa* (ATCC® 9027™) and *Acinetobacter baumannii* (ATCC® 19606™).

Figure S44. Dose response curve of compounds **1-9** against *Aspergillus fumigatus* (ATCC® 46645™).

Figure S45. Dose response curve of compounds **1-9** against A549 Human lung carcinoma cells (ATCC® CCL-185™).

Table S1. NMR spectral data of **3** and **4**.

Residue	Position	**3**		**4**	
		^{13}C	^{1}H, mult. (J = Hz)	^{13}C	^{1}H, mult. (J = Hz)
Proline (Pro)	α	58.6, CH	4.25, m	58.5, CH	4.24, m
	β	27.9, CH_2	1.92, m; 2.19, m	27.9, CH_2	1.90, m; 2.19, m
	γ	22.1, CH_2	1.88, m; 1.91, m	22.1, CH_2	1.84, m; 1.91, m
	δ	44.6, CH_2	3.40, m; 3.51, m	44.8, CH_2	3.41, m; 3.51, m
	CO	169.4, C	-	169.3, C	-
Oxazoline (Oxa)	α	53.9, CH	4.55, m	53.9, CH	4.55, m
	β	63.2, CH_2	4.54, m; 4.68, m	63.2, CH_2	4.54, m; 4.68, dd (16.0, 8.0)
	CN	160.5, C	-	160.1, C	-
	CO	163.2, C	-	163.2, C	-
Thiazole A	2	167.9, C	-	167.9, C	-
	4	147.2, C	-	147.2, C	-
	5	132.5, CH	8.76, s	132.8, CH	8.75, s
Pyridine (Py)	2	150.1, C	-	150.1, C	-
	3	127.77, C	-	127.6, C	-
	4	141.2, CH	8.44, d (8.2)	141.3, CH	8.45, d (8.2)
	5	118.3, CH	8.27, d (8.2)	118.4, CH	8.28, d (8.2)
	6	150.0, C	-	150.0, C	-
Thiazole B	2	160.5, C	-	160.3, C	-
	4	153.2, C	-	153.2, C	-
	5	122.8, CH	8.31, s	123.1, CH	8.31, s
Thiazole C	2	170.7, C	-	171.0, C	-
	4	146.4, C	-	146.7, C	-
	5	116.4, CH	7.37, s	116.3, CH	7.37, s
Phenylserine (PheSer)	α	58.1, CH	5.24, m	58.0, CH	5.24, m
	β	73.3, CH	5.03, m	73.6, CH	5.02, m
	1	141.1, C	-	141.6, C	-
	2,6	126.5, CH	7.29, (2H, m)	126.6, CH	7.31, (2H, m)
	3,5	127.80, CH	7.29, (2H, m)	127.8, CH	7.31, (2H, m)
	4	127.4, CH	7.24, m	127.5, CH	7.25, m
	NH	-	9.00, d (7.7)	-	9.01, d (8.0)
	OH	-	6.03, d (4.3)	-	6.03, d (4.5)
Glycine (Gly)	α	41.0, CH_2	3.88, dd (16.6, 4.0); 4.25, m	41.0, CH_2	3.81, dd (17.2, 4.0); 4.30 dd (17.2, 8.6)
	CO	169.6, C		169.4, C	-
	NH	-	8.56, m	-	8.46, m
Thiazole D	2	168.2, C	-	165.4, C	-
	4	148.0, C	-	143.7, C	-
	5	124.4, CH	8.29, s	140.6, C	-
	5-CH_2	-	-	67.2, CH_2	4.98, s
	5-CH_2O**Me**	-	-	58.4, CH_3	3.39, s
	CO	160.4, C	-	161.2, C	-

Valine (Val)	α	55.4, CH	5.29, dd (8.0, 4.9)	55.5, CH	5.26, s
	β	33.8, CH	2.22, m	33.9, CH	2.21, s
	γ	18.5, CH_3	0.87, d (6.9)	18.4, CH_3	0.86, d (6.6)
	γ'	17.7, CH_3	0.89, d (7.0)	17.7, CH_3	0.89, d (6.8)
	NH	-	8.76, d (8.0)	-	8.70, d (8.4)
Thiazole E	2	173.0, C	-	173.0, C	-
	4	148.4, C	-	148.6, C	-
	5	123.4, CH	8.10, s	123.3, CH	8.08, s
	CO	159.0, C	-	159.6, C	-
Asparagine (Asn)	α	48.0, CH	5.42, m	48.2, CH	5.37, m
	β	37.5, CH_2	1.50, m; 2.76, m	37.7, CH_2	1.35, m; 2.74, dd (16.5, 3.7)
	**CO**NHMe	169.3, C	-	169.5, C	-
	**NH**Me	-	7.48, m	-	7.40, m
	Me	25.3, CH_3	2.46, d (4.6)	25.6, CH_3	2.46, d (4.7)
	NH	-	8.81, d (9.0)	-	8.74, d (8.0)
Thiazole F	2	164.5, C	-	164.6, C	-
	4	149.3, C	-	149.2, C	-
	5	126.8, CH	8.61, s	126.9, CH	8.62, s
	CO	160.0, C	-	160.2, C	-

^{1}H (400 MHz) and ^{13}C (100 MHz) in DMSO-d_6. Assignments based on COSY, HSQC and HMBC and comparison with literature compounds. Chemical shifts (δ) in ppm. s: singlet; br s: broad singlet; d: doublet; br d: broad doublet; t: triplet, m: multiplet. One proton unless otherwise stated.

Table S2. ^{1}H and ^{13}C NMR data of **5** and **6.**

Residue	Position	**5**		**6**	
		^{13}C	^{1}H, mult. (J = Hz)	^{13}C	^{1}H, mult. (J = Hz)
Proline (Pro)	α	59.8, CH	4.33, dd (8.6, 3.8)	59.8, CH	4.33, dd (8.8, 3.6)
	β	29.1, CH_2	2.08, m; 1.86, m	29.3, CH_2	1.87, m; 2.09, m
	γ	24.4, CH_2	1.92, (2H, m)	24.4, CH_2	1.92, (2H, m)
	δ	47.0, CH_2	3.77, (2H, m)	47.1, CH_2	3.77, (2H, m)
	CO	173.5, C	-	173.6, C	
Serine (Ser)	α	53.0, CH	4.90, dd (12.0, 4.0)	53.0, CH	4.90, dd (12.0, 4.0)
	β	61.7, CH_2	3.76, m; 3.82, m	61.8, CH_2	3.80, m; 3.84, m
	CO	168.9	-	168.9, C	-
	NH	-	8.47, m	-	8.47, m
	OH	-	5.28, br t (6.0)	-	5.28, m
Thiazole A	2	167.3, C	-	167.3, C	-
	4	150.8, C	-	150.7, C	-
	5	127.6, CH	8.55, s	127.5, CH	8.55, s
	CO	160.1, C	-	160.1, C	-
Pyridine (Py)	2	150.3, C	-	150.2, C	-
	3	127.7, C	-	127.6, C	-
	4	141.3, CH	8.44, d (7.0)	141.2, CH	8.46, d (6.8)
	5	118.5, CH	8.40, d (7.0)	118.6, CH	8.41, d (6.8)
	6	150.1, C	-	150.0, C	-
Thiazole B	2	160.4, C	-	160.3, C	-
	4	153.1, C	-	153.2, C	-
	5	123.0, CH	8.30, s	123.0, CH	8.30, s
Thiazole C	2	170.9, C	-	171.0, C	-
	4	146.7, C	-	146.7, C	-
	5	116.3, CH	7.36, s	116.3, CH	7.36, s
Phenylserine (PheSer)	α	58.0, CH	5.24, m	58.1, CH	5.24, m
	β	73.5, CH	5.03, br t (4.0)	73.7, CH	5.01, dd (6.2, 5.0)
	1	141.6, CH	-	141.7, C	-
	2,6	126.6, CH	7.30, (2H, m)	126.6, CH	7.31, m
	3,5	127.8, CH	7.27, (2H, m)	127.8, CH	7.30, m
	4	127.8, CH	7.25, m	127.7, CH	7.25, m
	NH	-	9.01, d (8.3)	-	9.01, d (7.8)
	OH	-	6.02, d (4.5)	-	6.03, d (4.6)
Glycine (Gly)	α	41.4, CH_2	3.87, m; 4.21, dd (16.6, 7.9)	41.1, CH_2	3.79, m; 4.26, dd (17.5, 8.6)
	CO	169.3, C	-	169.3, C	-
	NH	-	8.55, m	-	8.44, m
Thiazole D	2	168.4, C	-	165.5, C	-
	4	148.3, C	-	143.6, C	-
	5	124.5, CH	8.28, s	140.8, C	-
	5-CH_2	-	-	67.3, CH_2	4.98, s
	5-CH_2O**Me**	-	-	58.5, CH_3	3.38, s
	CO	161.0, C	-	161.2, C	-

Valine (Val)	α	55.4, CH	5.24, m	55.2, CH	5.20, m
	β	34.0, CH	2.20, m	34.0, CH	2.18, m
	γ	18.4, CH_3	0.86, d (8.0)	18.3, CH_3	0.85, d (6.8)
	γ'	17.7, CH_3	0.88, d (8.0)	17.8, CH_3	0.88, d (6.8)
	NH	-	8.73, d (7.8)		8.67, d (8.2)
Thiazole E	2	167.9, C	-	168.3, C	-
	4	141.7, C	-	141.9, C	-
	5	139.6, C	-	139.3, C	-
	5-Me	11.8, CH_3	2.60, s	11.8, CH_3	2.59, s
	CO	160.3, C	-	159.8, C	-
Asparagine (Asn)	α	47.9, CH	5.37, m	47.9, CH	5.31, m
	β	37.6, CH_2	1.50, m; 2.73, dd (16.4, 3.7)	37.5, CH_2	1.37, m; 2.72, dd (16.4, 3.2)
	**CO**NHMe	169.6, C	-	169.6, C	-
	**NH**Me	-	7.48, m	-	7.38, m
	Me	25.6, CH_3	2.46, d (4.6)	25.7, CH_3	2.47, d (4.5)
	NH	-	8.77, d (9.0)	-	8.70, d (8.7)
Thiazole F	2	164.7, C	-	164.7, C	-
	4	149.3, C	-	149.3, C	-
	5	126.8, CH	8.60, s	126.9, CH	8.61, s
	CO	159.8, C	-	161.1, C	-

^{1}H (400 MHz) and ^{13}C (100 MHz) in DMSO-d_6. Assignments based on COSY, HSQC and HMBC and comparison with literature compounds. Chemical shifts (δ) in ppm. s: singlet; br s: broad singlet; d: doublet; br d: broad doublet; t: triplet, m: multiplet. One proton unless otherwise stated.

Table S3. ^{1}H and ^{13}C NMR data of **7** and ^{1}H NMR data of **8** and **9**.

Residue	Position	**7**		**8**	**9**
		^{13}C	^{1}H, mult. (J = Hz)	^{1}H, mult. (J = Hz)	^{1}H, mult. (J = Hz)
Thiazole A	2	168.1, C	-	-	-
	4	147.3, C	-	-	-
	5	132.3, CH	8.78, s	8.79, s	8.79, s
	CO	161.1, C	-	-	-
	COO**Me**	52.2 CH_3	3.91, s	3.91, s	-
Pyridine (Py)	2	150.1, C	-	-	-
	3	127.6, C	-	-	-
	4	141.3, CH	8.41, d (8.2)	8.43, d (8.1)	8.43, d (8.2)
	5	118.5, CH	8.30, d (8.2)	8.30, d (8.1)	8.30, d (8.2)
	6	150.0, C	-	-	-
Thiazole B	2	160.4, C	-	-	-
	4	153.1, C	-	-	-
	5	123.0, CH	8.29, s	8.29, s	8.29, s
Thiazole C	2	170.8, C	-	-	-
	4	146.6, C	-	-	-
	5	116.3, CH	7.32, s	7.33, s	7.34, s
Phenylserine (PheSer)	α	58.3, CH	5.23, m	5.20, m	5.20, m
	β	73.2, CH	5.06, m	5.04, m	5.03, m
	1	141.6, C	-	-	-
	2,6	126.5, CH	7.26 (2H, m)	7.28 (2H, m)	7.29, m
	3,5	127.7, CH	7.25 (2H, m)	7.27 (2H, m)	7.28, m
	4	127.5, CH	7.21, m	7.22, m	7.22, m
	NH	-	9.46, d (7.6)	9.45, d (6.2)	9.29, d (6.6)
	OH	-	6.70, br s	6.65, br s	6.44, br s
Glycine (Gly)	α	41.3, CH_2	3.89, dd (17.2, 4.0); 4.29, dd (17.2, 8.9)	3.81, m; 4.34, m	3.80, dd (16.7, 3.6); 4.31, dd (16.7, 8.6)
	CO	169.4, C	-	-	8.45, m
	NH	-	8.57, m	8.46, m	-
Thiazole D	2	168.3, C	-	-	-
	4	148.4, C	-	-	-
	5	124.5, CH	8.28, s	-	-
	5-CH_2	-	-	4.99, s	4.98, s
	5-CH_2O**Me**	-	-	3.39	3.39, s
	CO	161.1, C	-	-	-
Valine (Val)	α	55.4, CH	5.24, m	5.23, m	5.24, m
	β	34.0, CH	2.19, m	2.18, m	2.18, m
	γ	18.4, CH_3	0.86, d (6.8)	0.89, d (6.8)	0.88, d (6.6)
	γ'	17.8, CH_3	0.89, d (6.8)	0.85, d (6.8)	0.85, d (6.8)
	NH	-	8.76, d (8.2)	8.70, d (8.3)	8.69, br s
Thiazole E	2	167.9, C	-	-	-
	4	141.7, C	-	-	-
	5	139.6, C	-	-	-
	5-Me	11.8, CH_3	2.60, s	2.59, s	2.59, s
	CO	160.5, C	-	-	-

Asparagine (Asn)	α	47.9, CH	5.34, m	5.29, m	5.30, dd (8.6, 4.6)
	β	37.6, CH_2	1.43, m; 2.75, m	1.29, m; 2.72, m	1.34, m; 2.72, dd (16.0, 3.4)
	**CO**NHMe	169.6, C	-	-	-
	**NH**Me	-	7.50, m	7.42, m	7.41, m
	Me	25.7, CH_3	2.45, d (4.5)	2.46, d (4.6)	2.47, d (4.6)
	NH	-	8.73, d (9.4)	8.68, d (8.8)	8.67, br s
Thiazole F	2	164.6, C	-	-	-
	4	149.3, C	-	-	-
	5	126.9, CH	8.61, s	8.61, s	8.61, br s
	CO	160.2	-	-	-

^{1}H (400 MHz) and ^{13}C (100 MHz) in DMSO-d_6. Assignments based on COSY, HSQC and HMBC and comparison with literature compounds. Chemical shifts (δ) in ppm. s: singlet; br s: broad singlet; d: doublet; br d: broad doublet; t: triplet, m: multiplet. One proton unless otherwise stated.

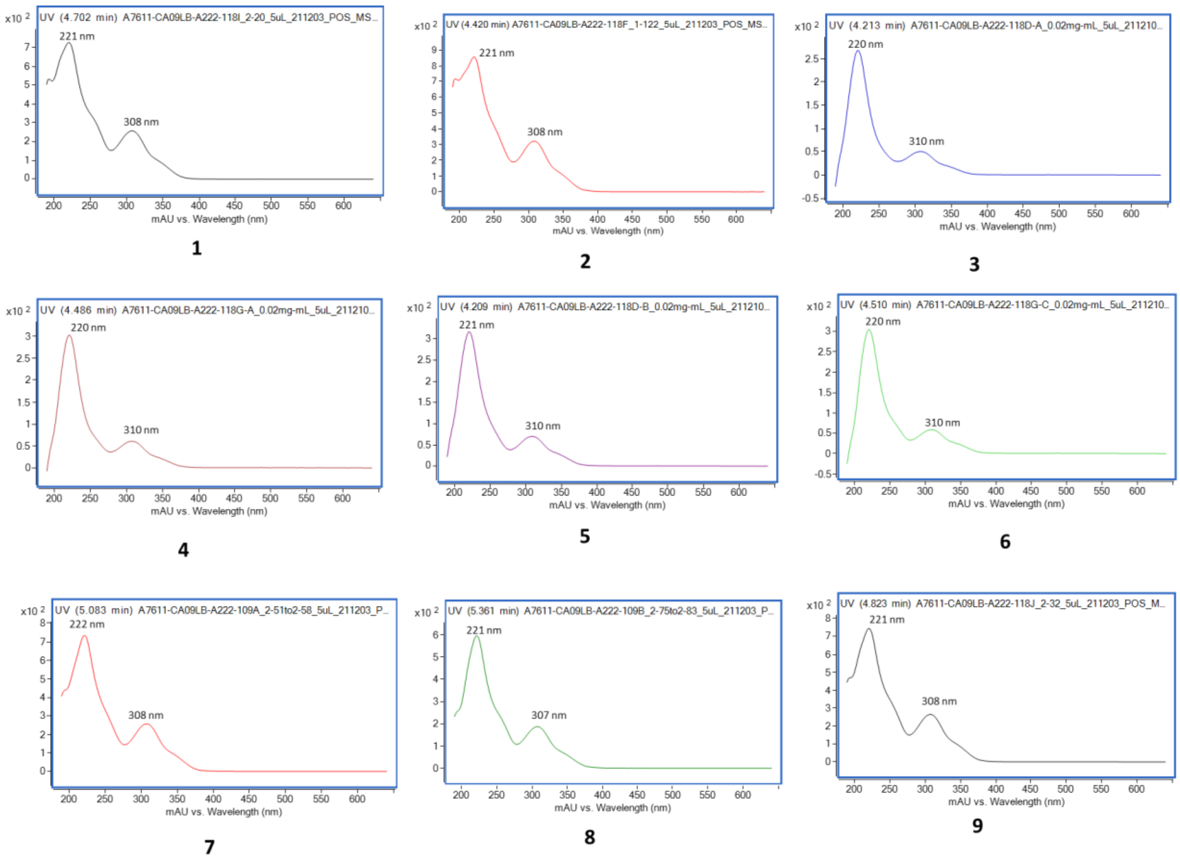

Figure S1. UV spectra for compounds **1**–**9**.

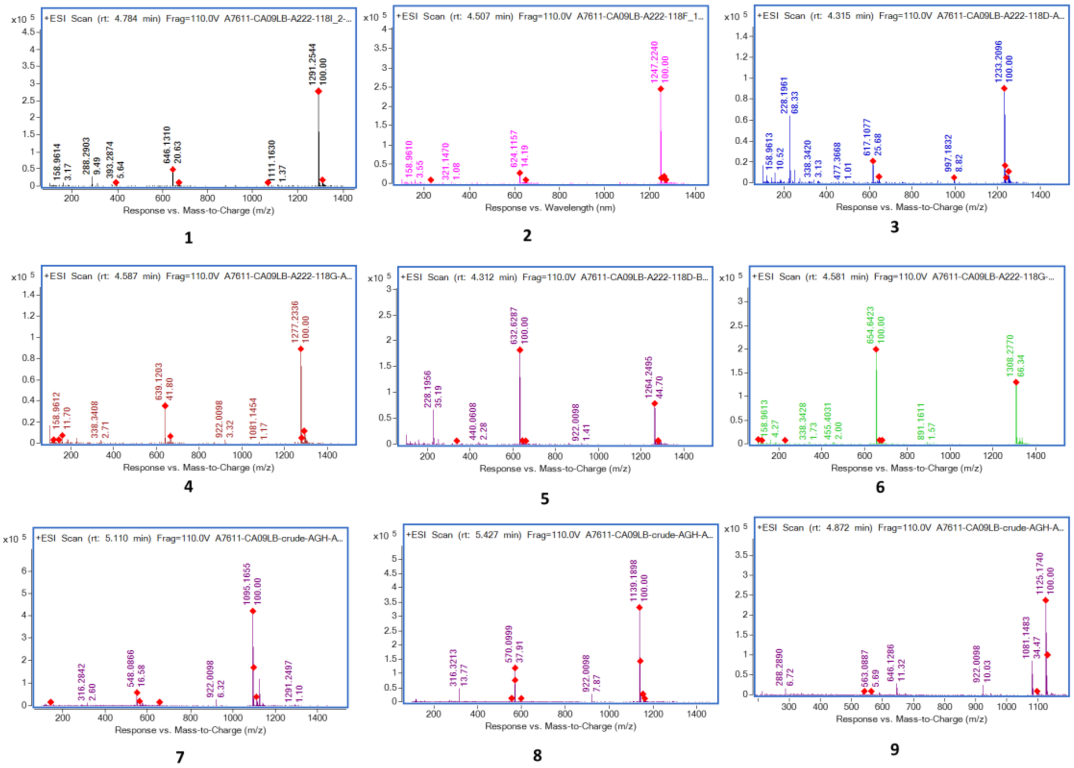

Figure S2. (+)-HRESIMS spectra for compounds **1**–**9**.

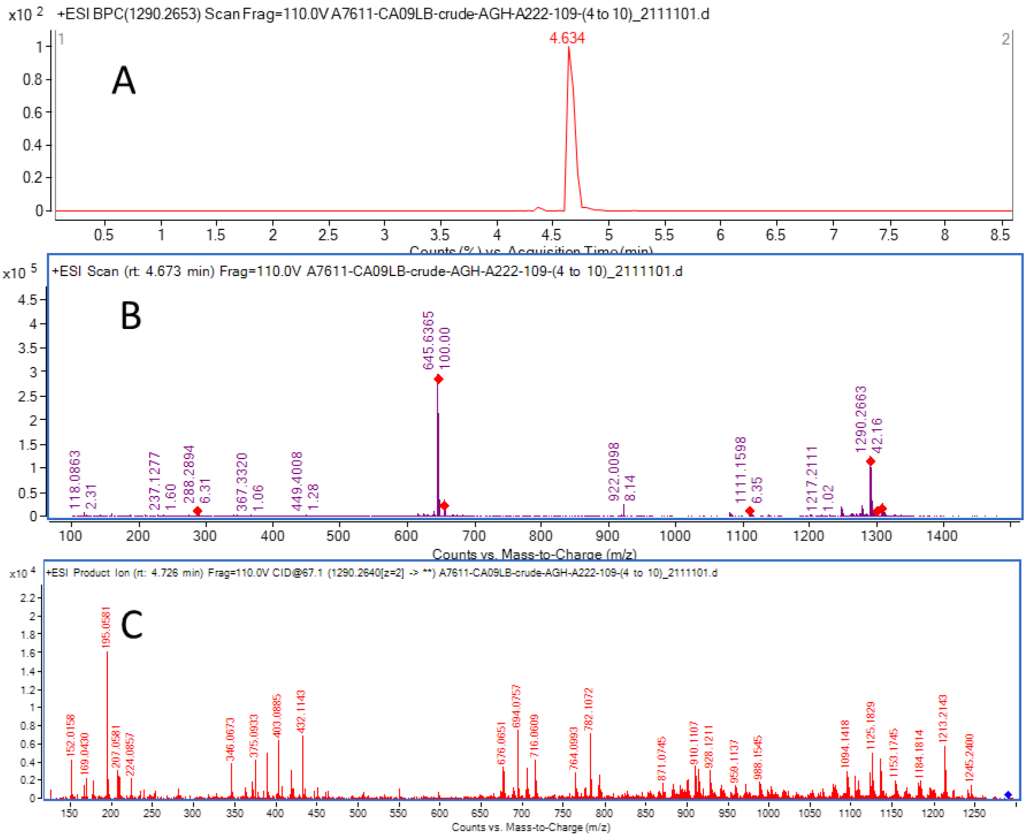

Figure S3. A. Extracted ion chromatogram (*m/z* 1290.2653 ± 5 ppm), **B.** (+)-HRESIMS spectrum, and **C**. MS/MS spectrum of GE2270A in the extract of *Nonomuraea jiangxiensis* strain A7611.

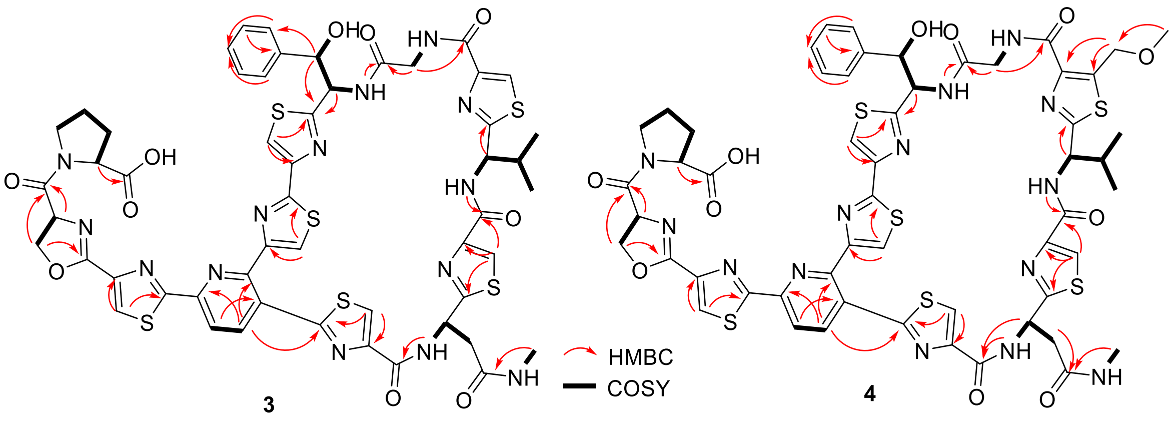

Figure S4. Selected COSY and HMBC correlations of **3** and **4**.

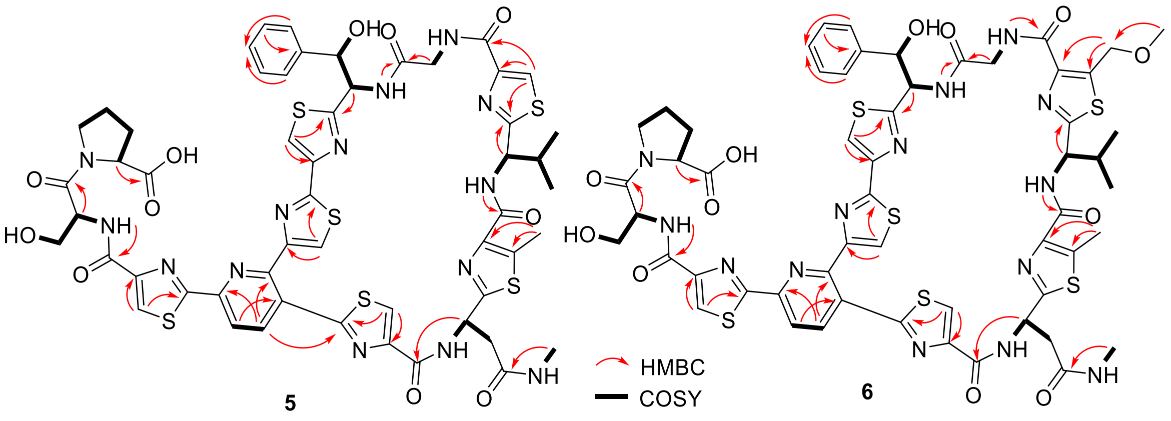

Figure S5. Selected COSY and HMBC correlations of **5** and **6**.

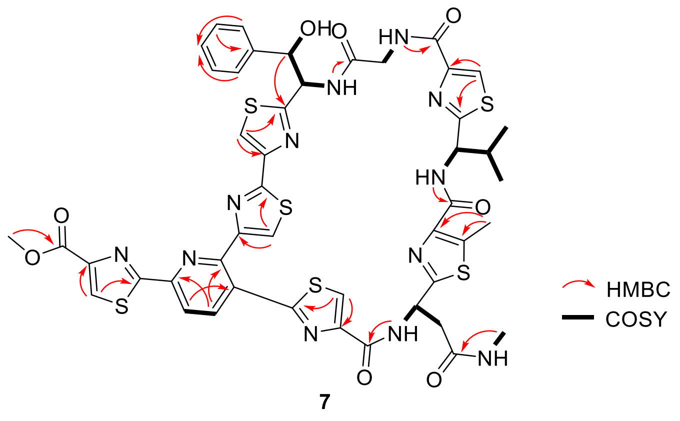

Figure S6. Selected COSY and HMBC correlations of **7**.

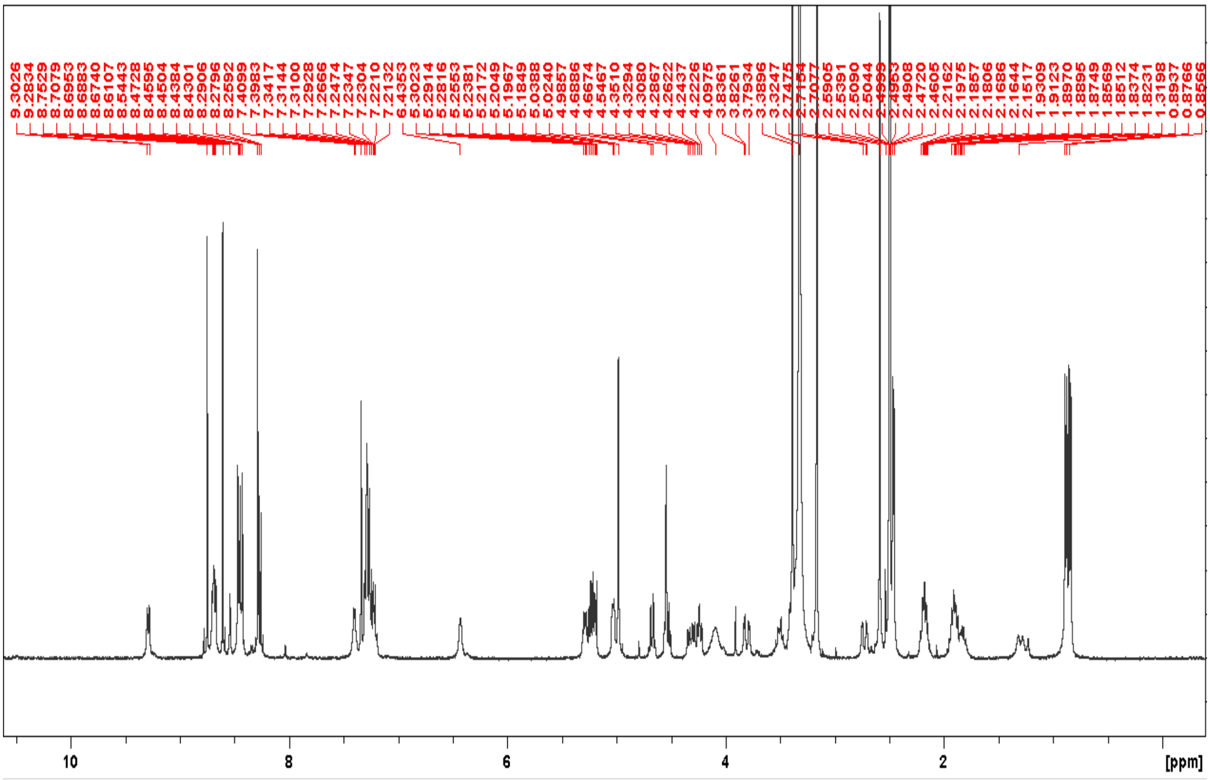

Figure S7. ^{1}H NMR spectrum (DMSO-d_6, 400 MHz) of **1**.

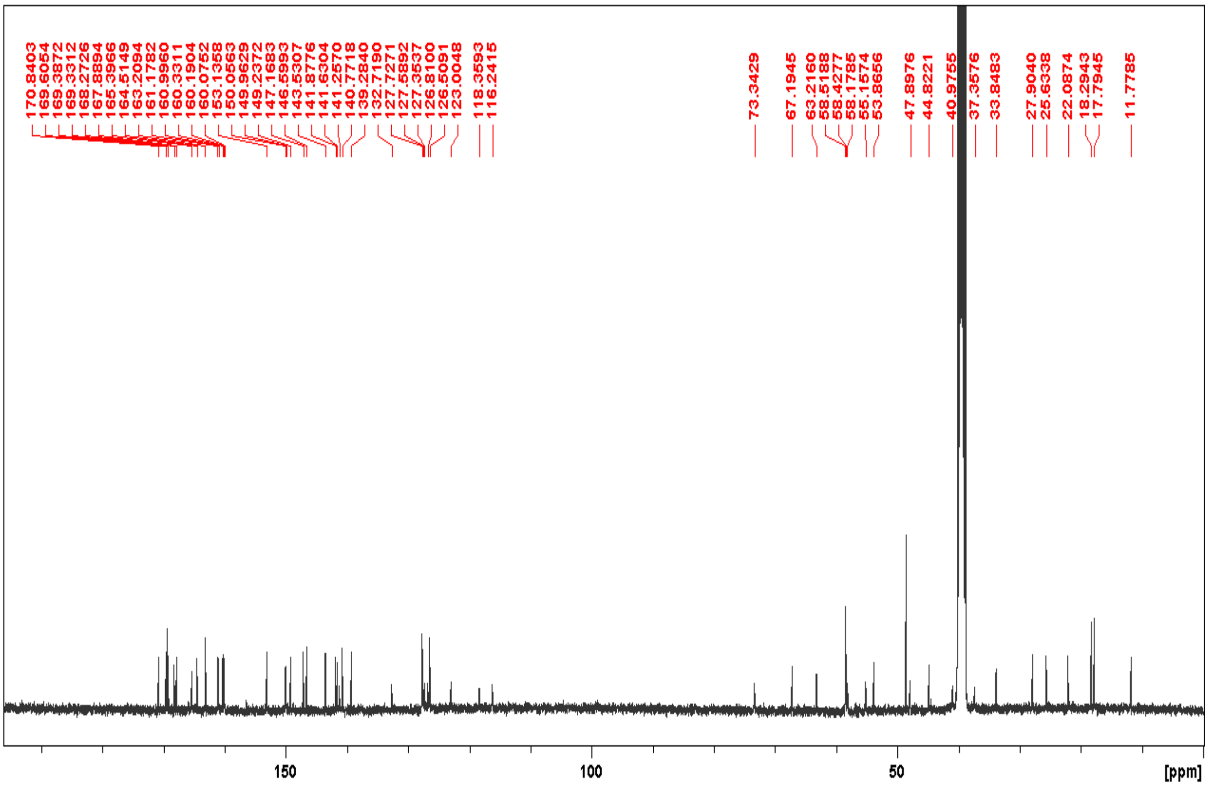

Figure S8. ^{13}C NMR spectrum (DMSO-d_6, 100 MHz) of **1**.

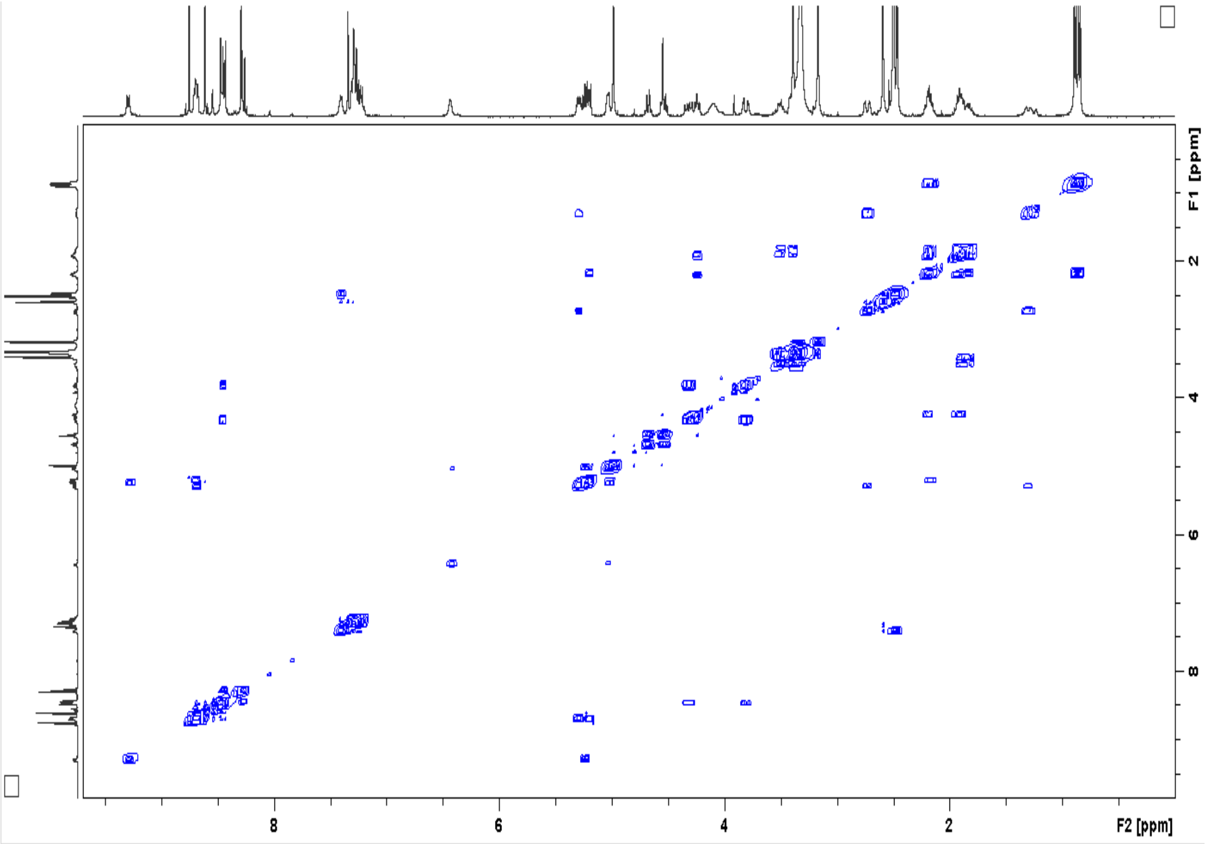

Figure S9. COSY spectrum of **1**.

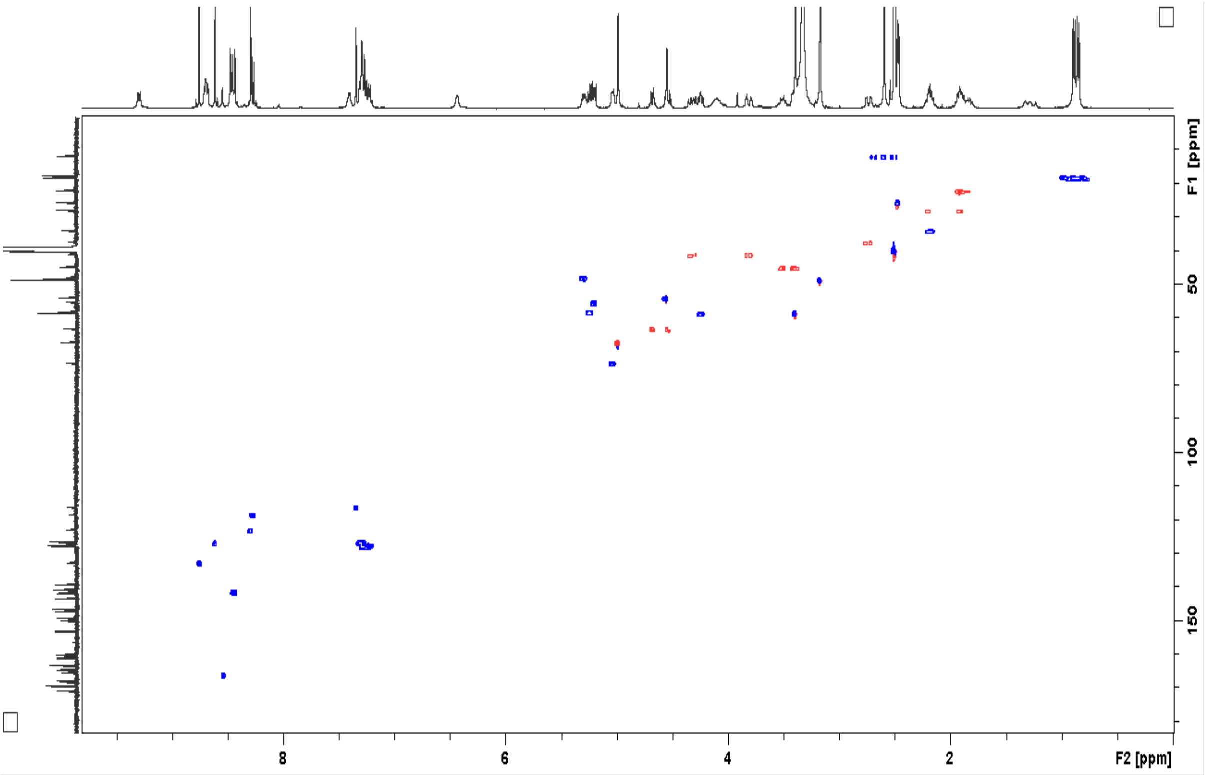

Figure S10. HSQC spectrum of **1**.

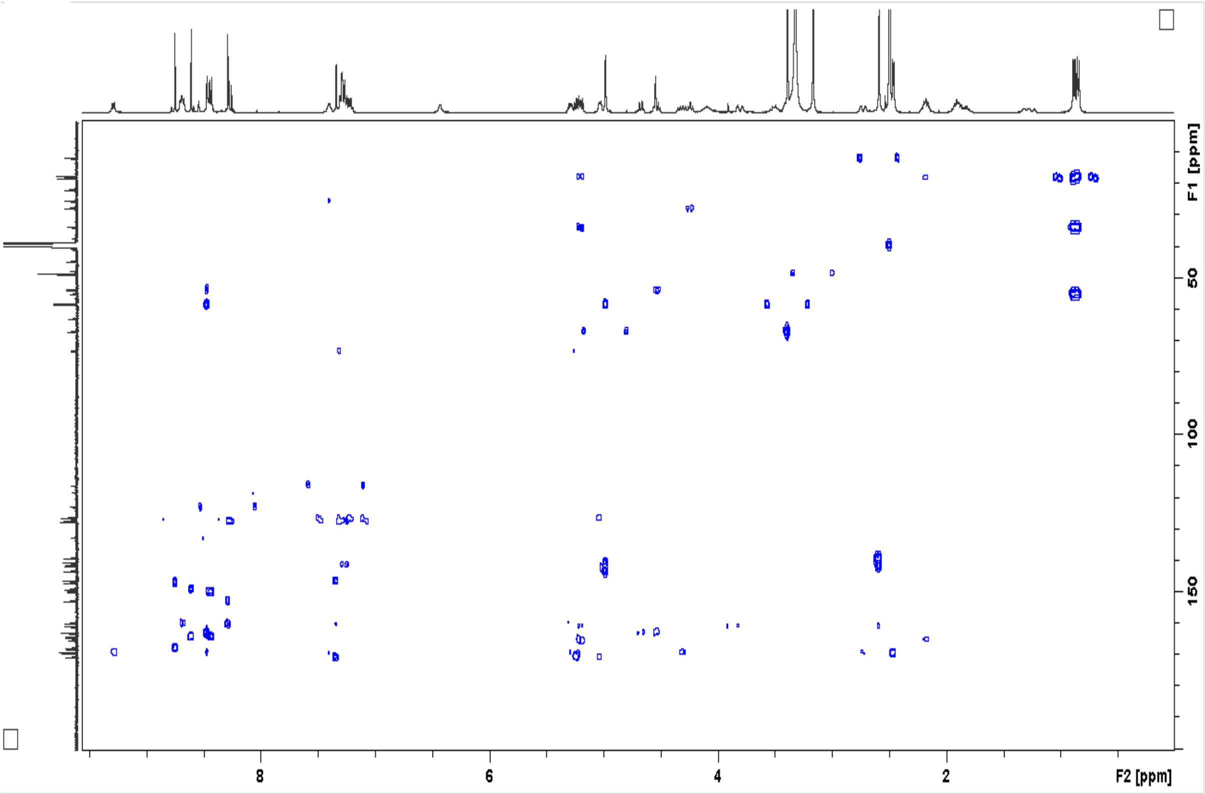

Figure S11. HMBC spectrum of **1**.

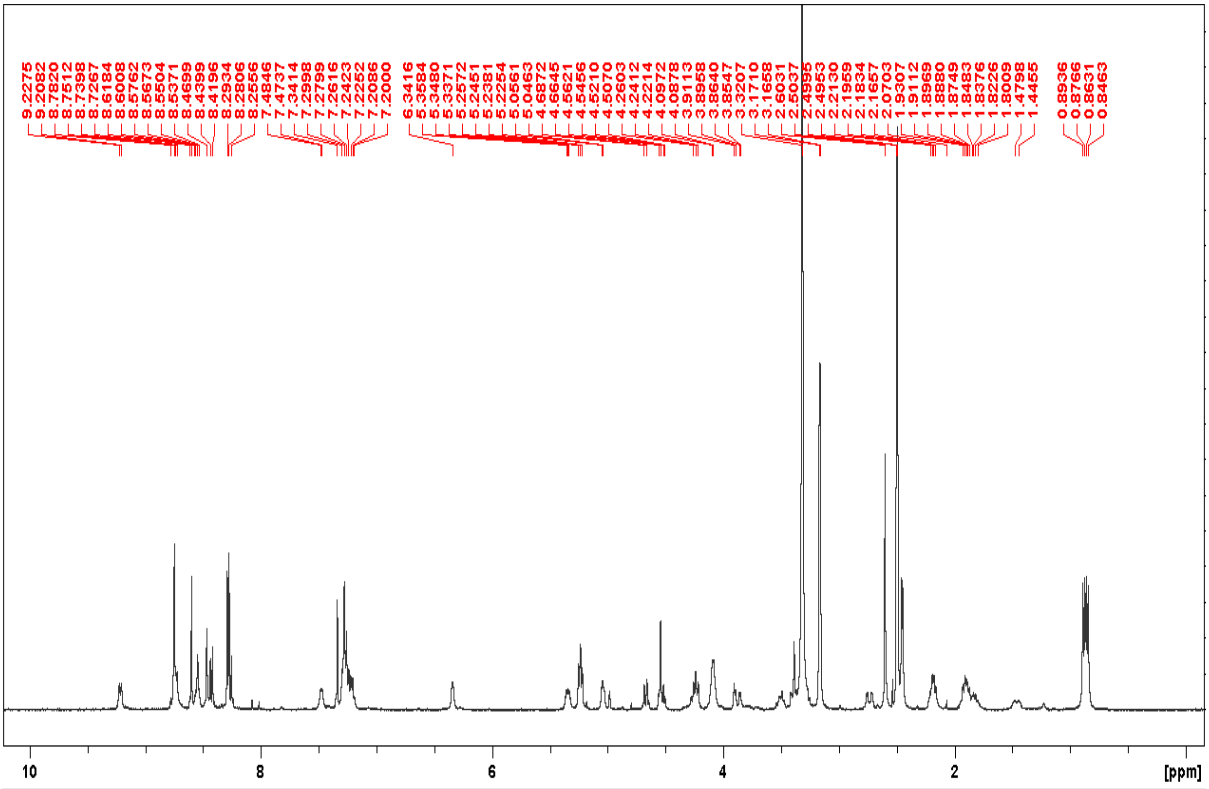

Figure S12. ^{1}H NMR spectrum (DMSO-d_6, 400 MHz) of **2**.

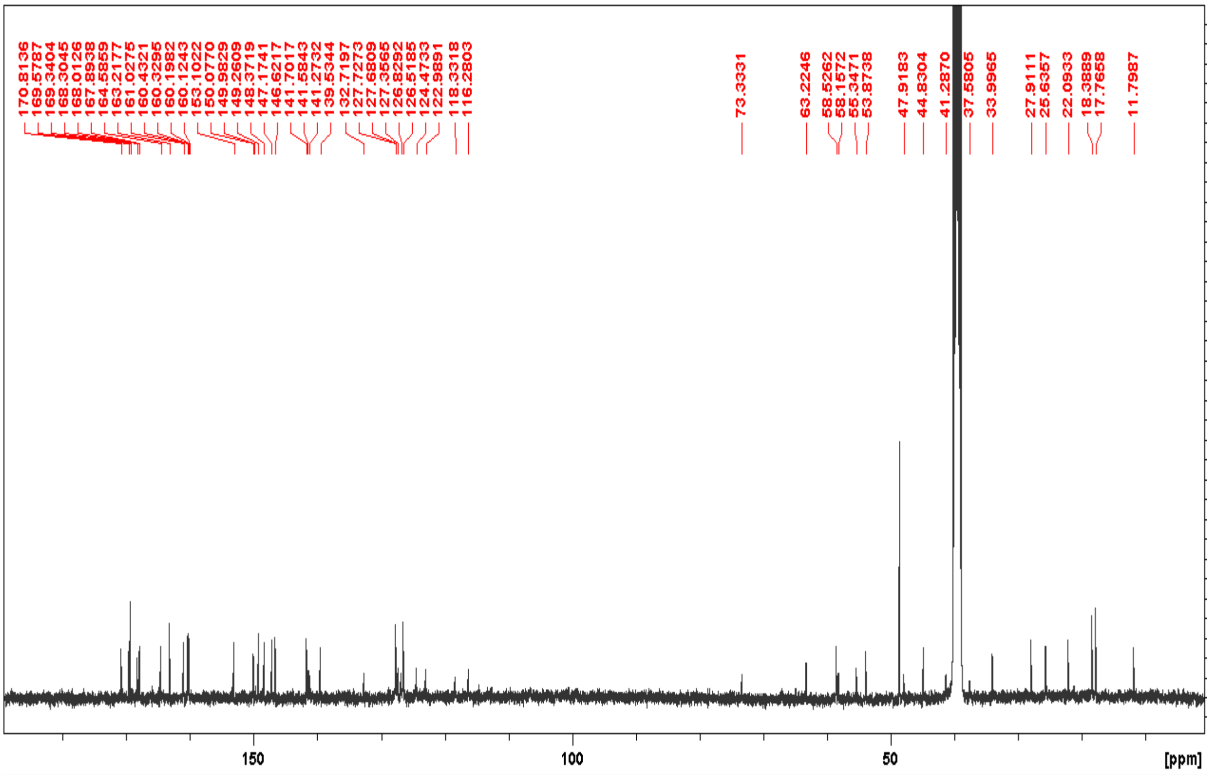

Figure S13. ^{13}C NMR spectrum (DMSO-d_6, 100 MHz) of **2**.

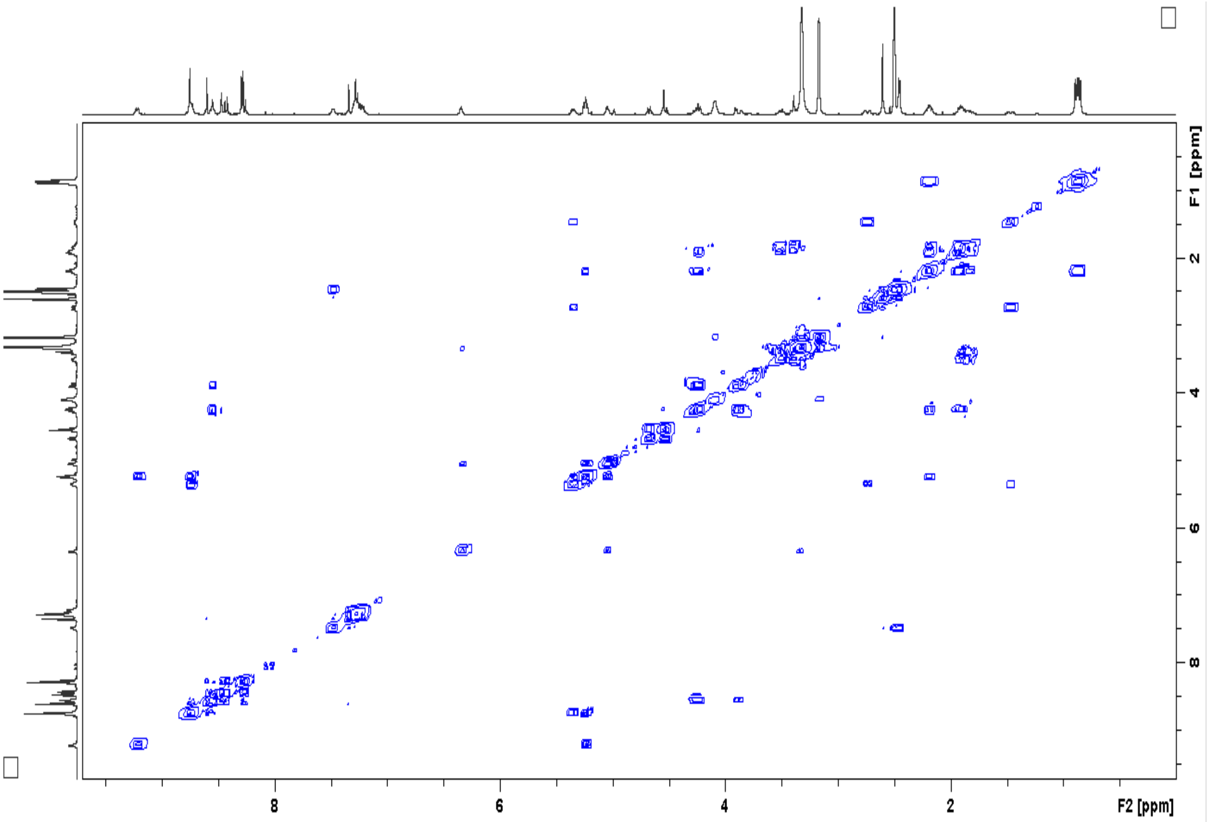

Figure S14. COSY spectrum of **2**.

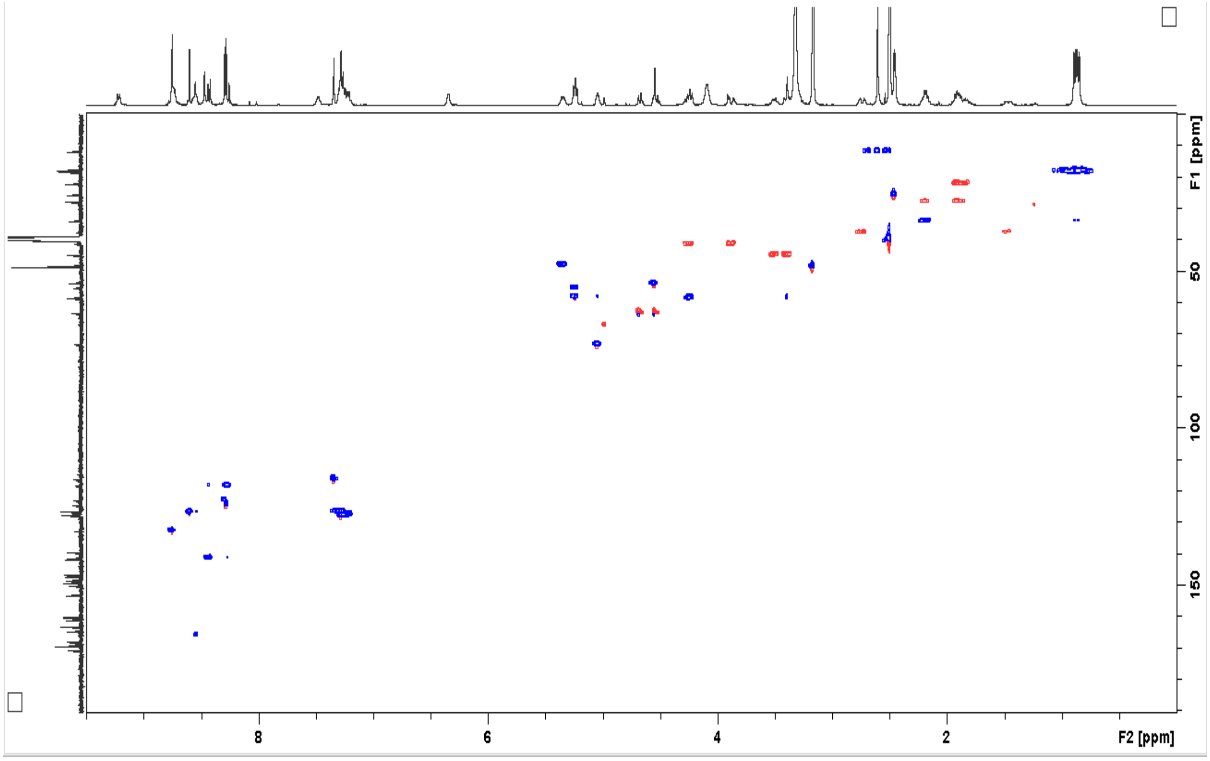

Figure S15. HSQC spectrum of **2**.

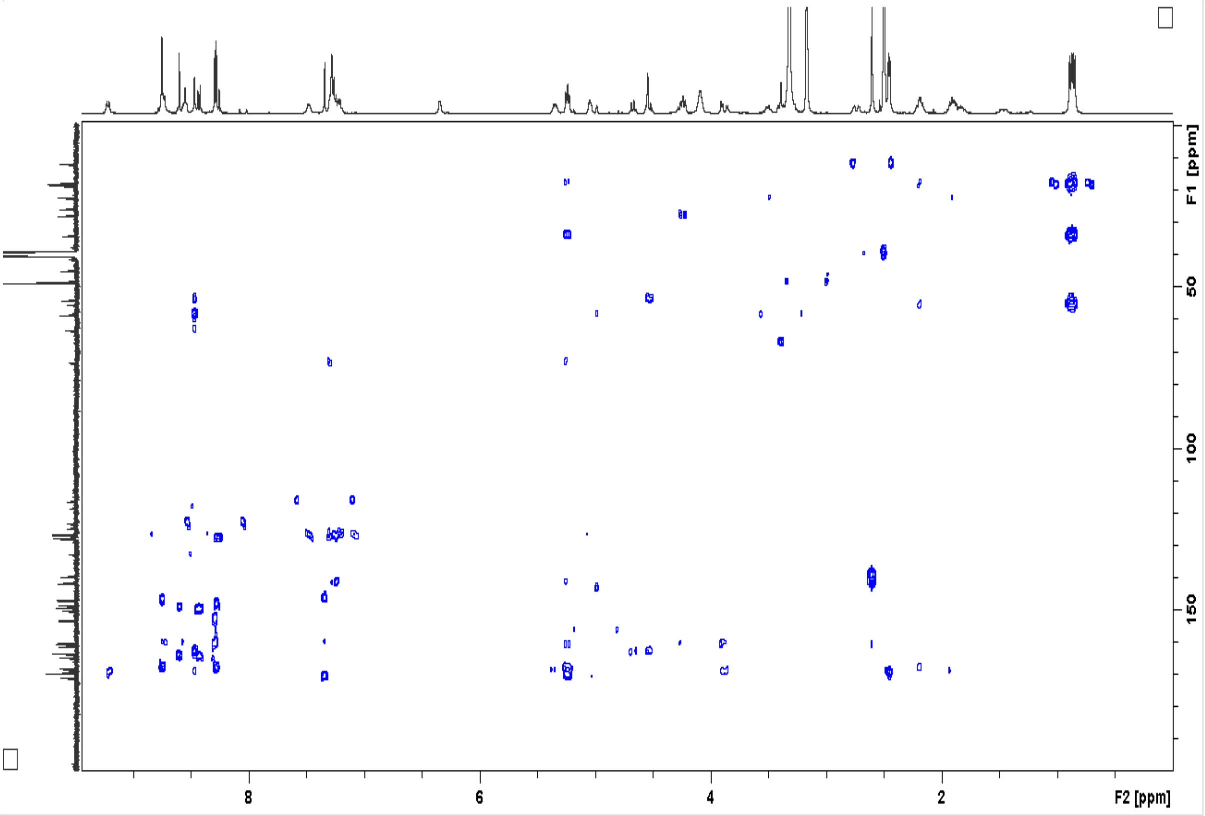

Figure S16. HMBC spectrum of **2**.

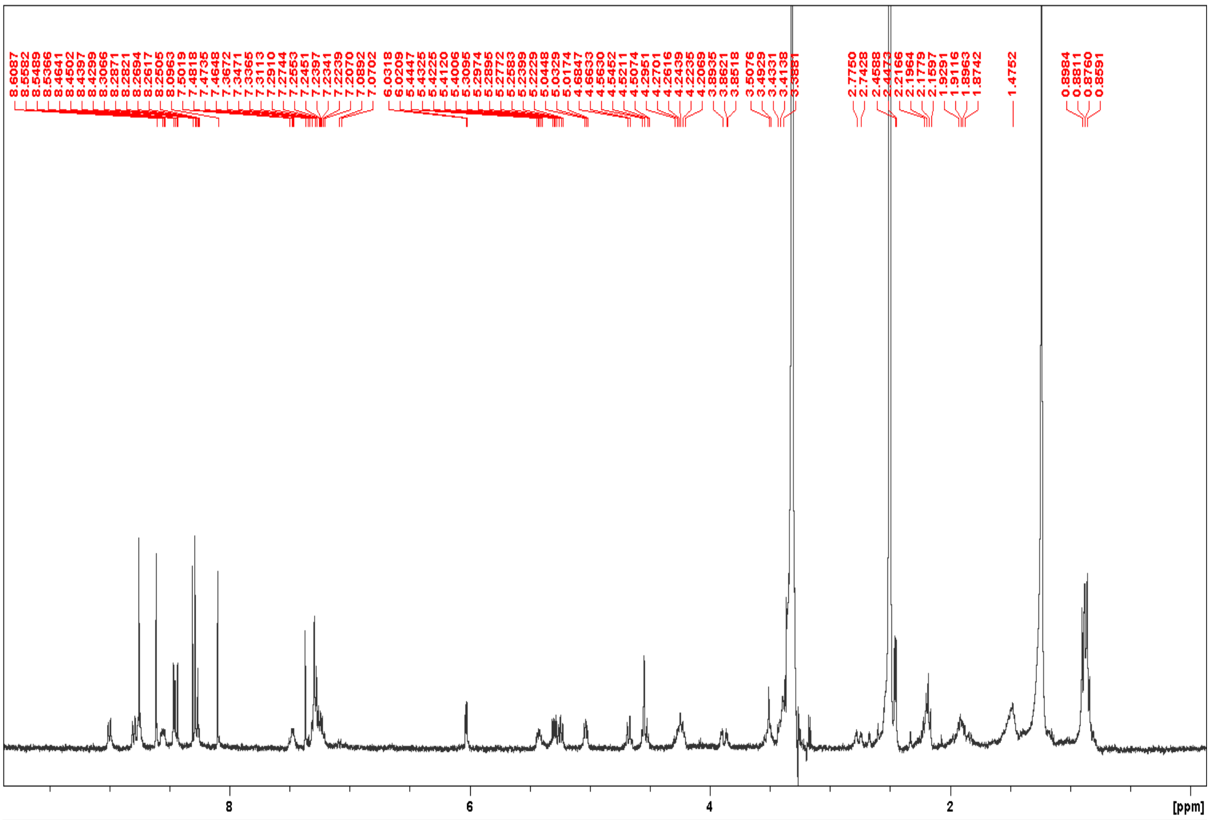

Figure S17. ^{1}H NMR spectrum (DMSO-d_6, 400 MHz) of **3**.

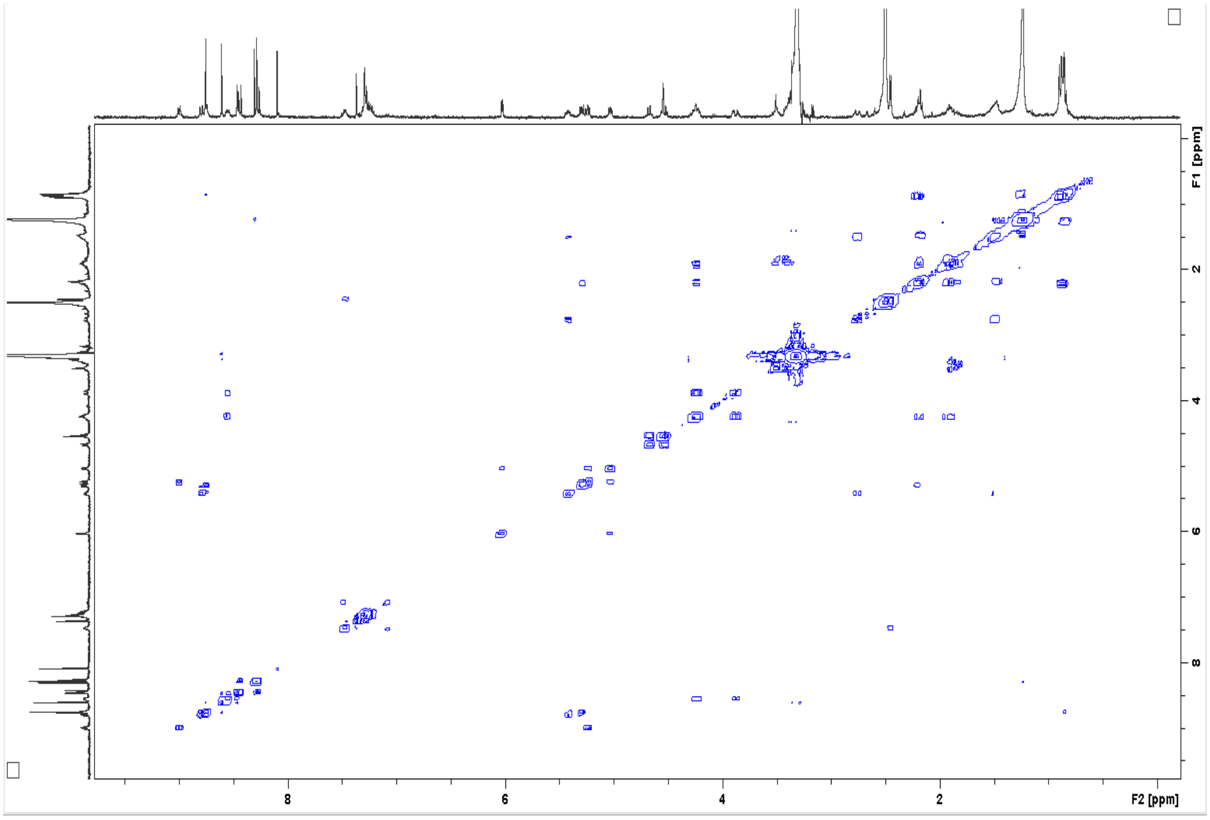

Figure S18. COSY spectrum of **3**.

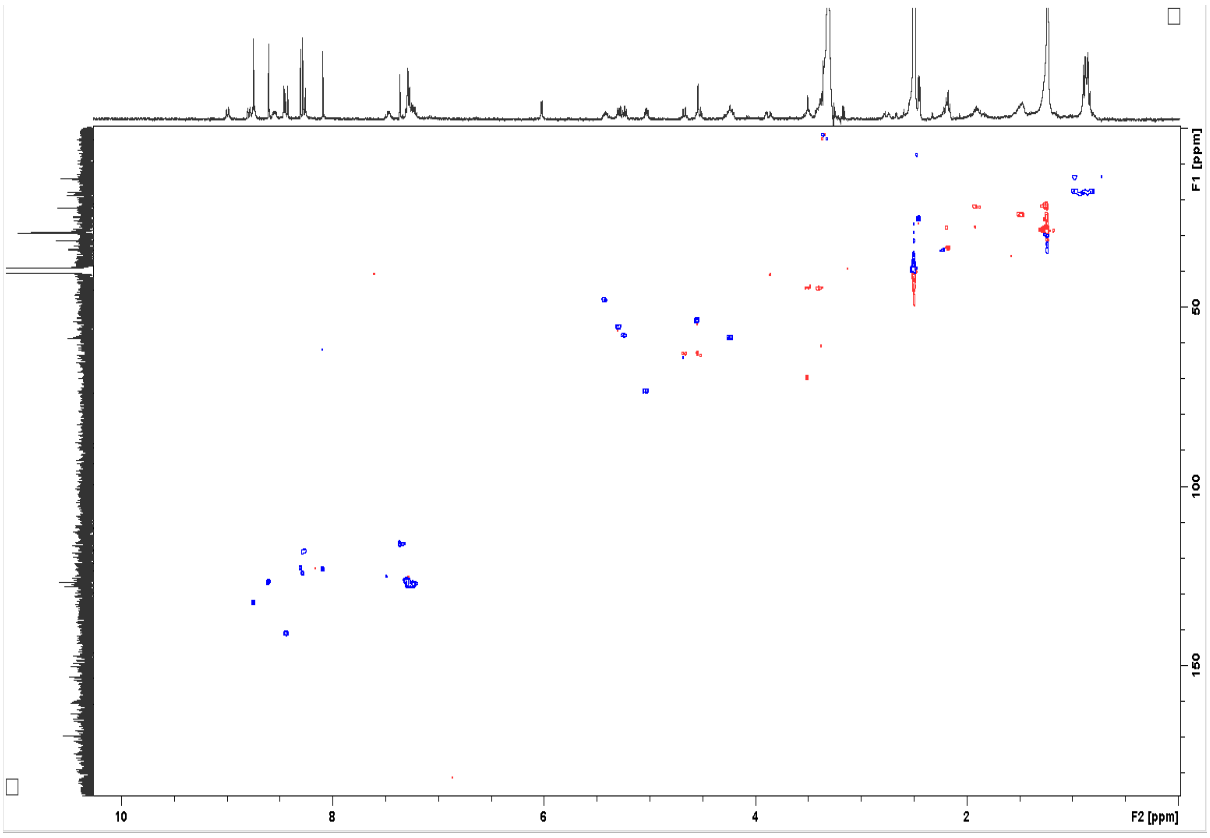

Figure S19. HSQC spectrum of **3**.

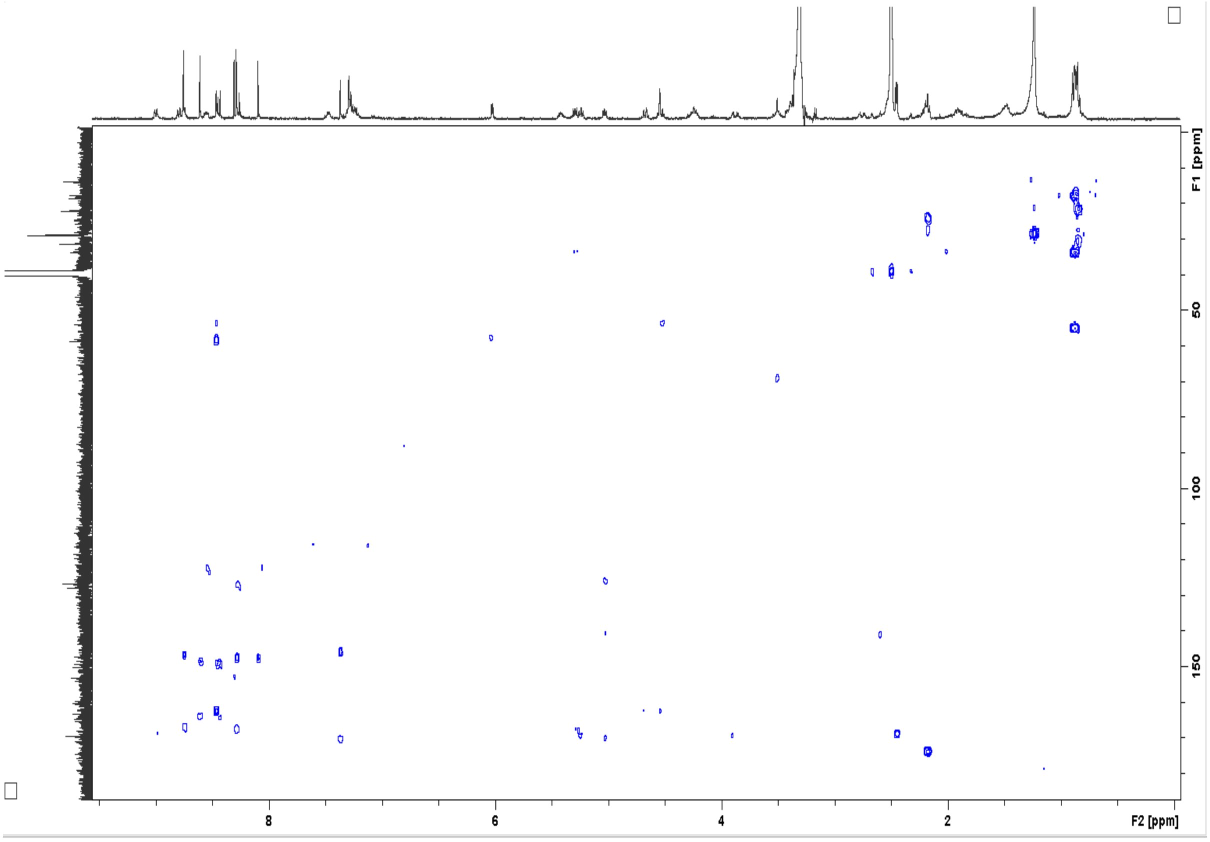

Figure S20. HMBC spectrum of **3**.

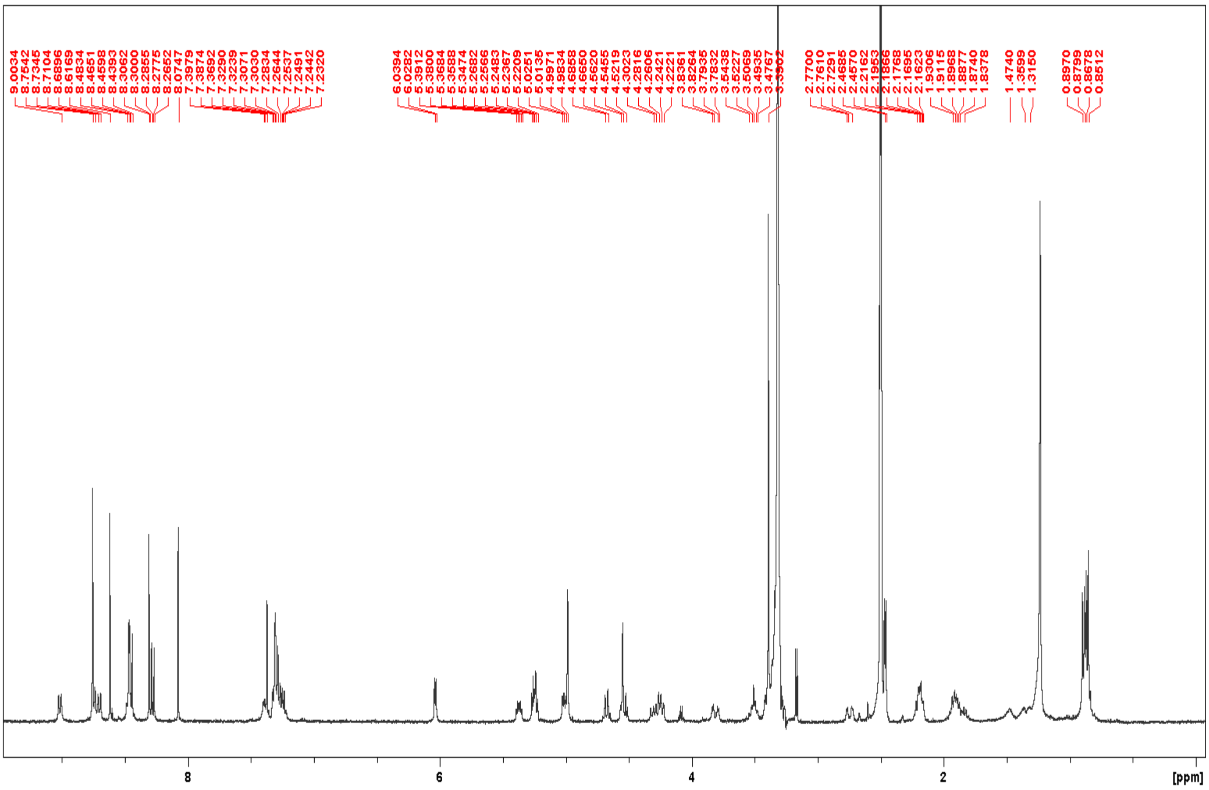

Figure S21. ^{1}H NMR spectrum (DMSO-d_6, 400 MHz) of **4**.

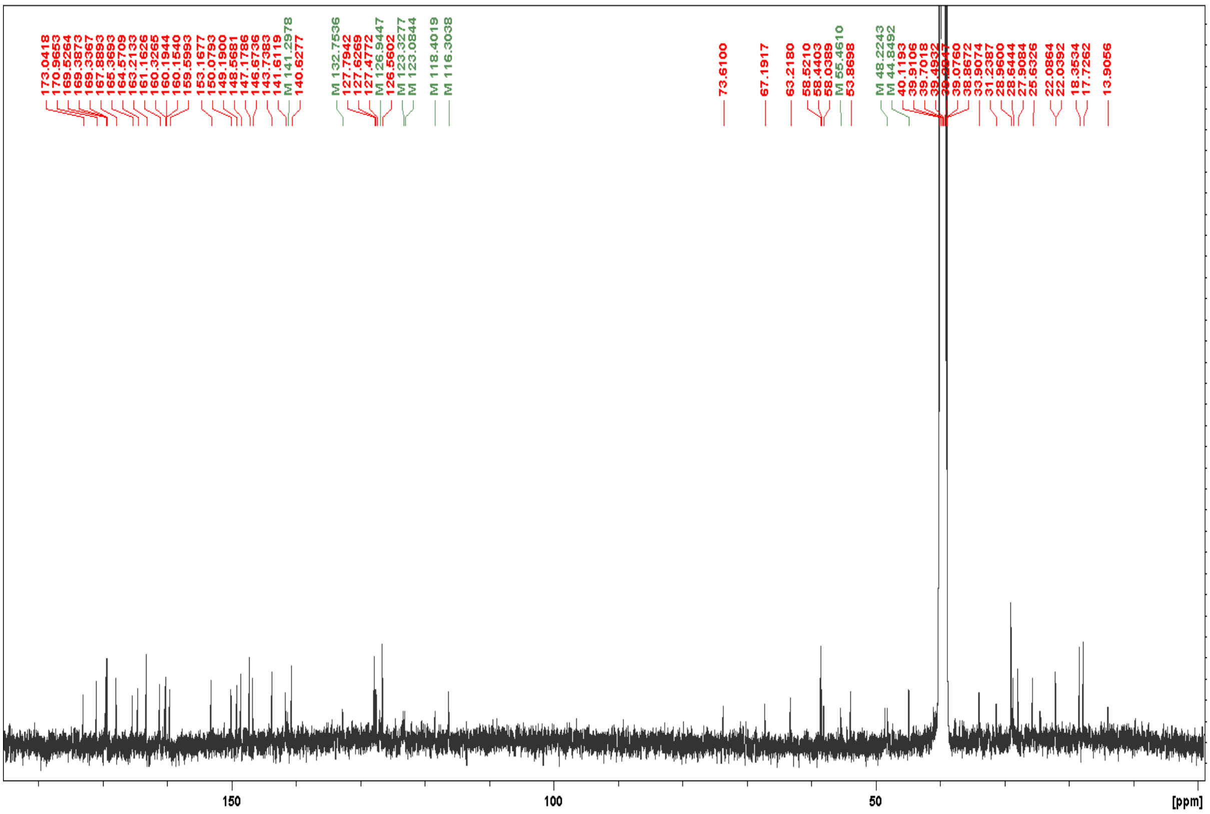

Figure S22. ^{13}C NMR spectrum (DMSO-d_6, 100 MHz) of **4**.

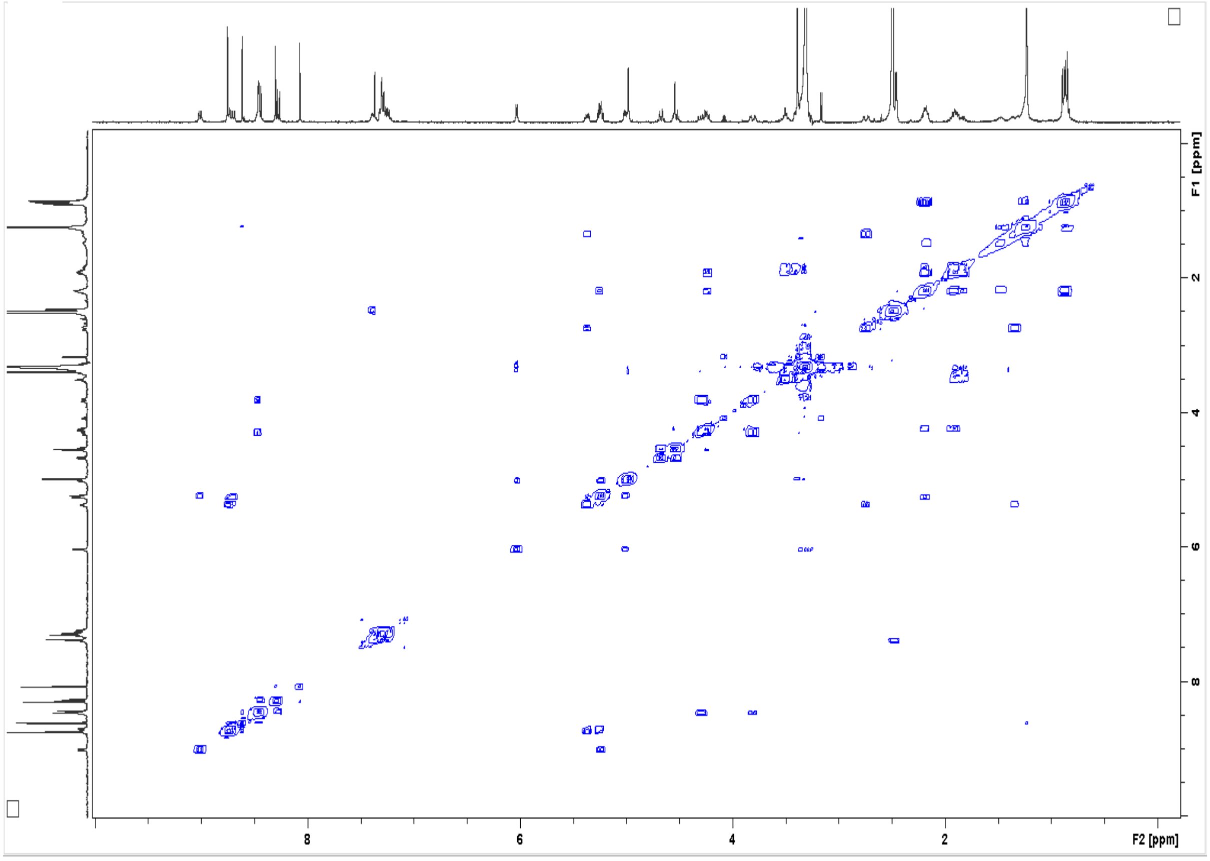

Figure S23. COSY spectrum of **4**.

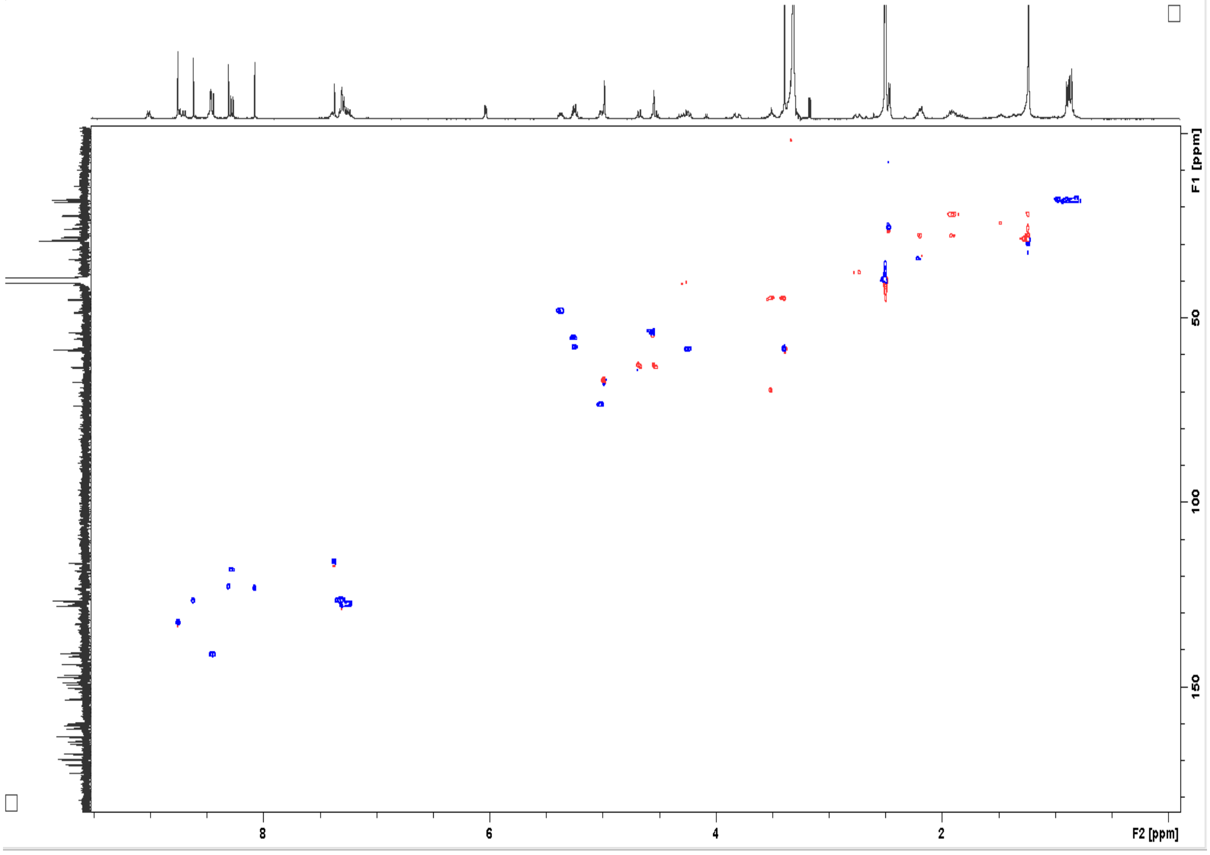

Figure S24. HSQC spectrum of **4**.

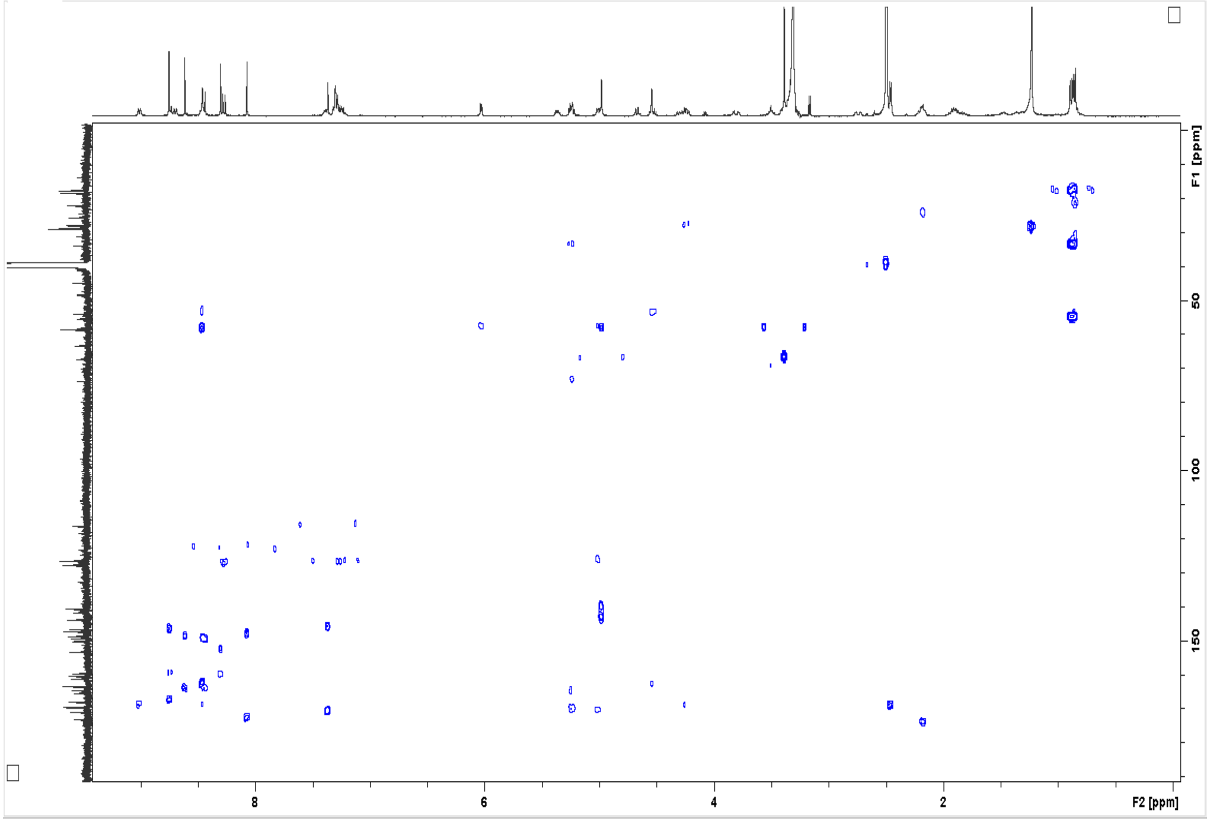

Figure S25. HMBC spectrum of **4**.

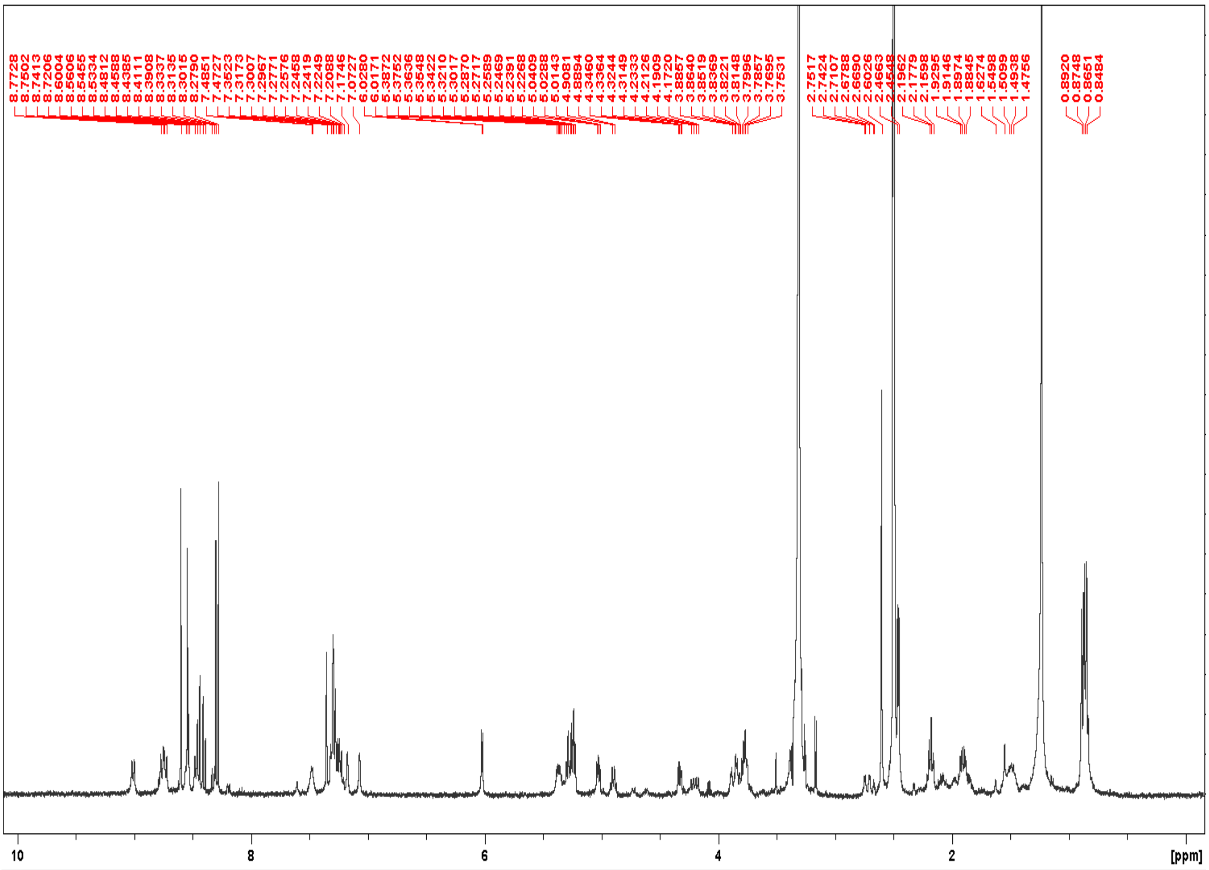

Figure S26. ^{1}H NMR spectrum (DMSO-d_6, 400 MHz) of **5**.

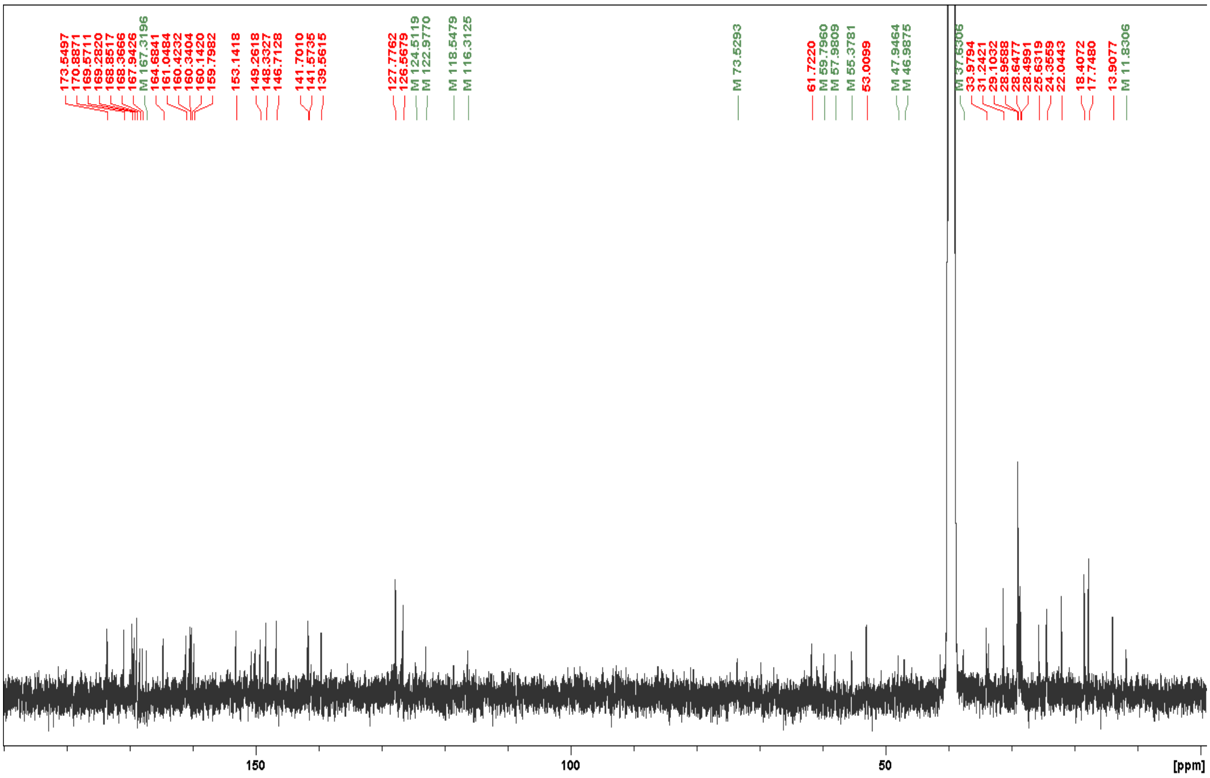

Figure S27. ^{13}C NMR spectrum (DMSO-d_6, 100 MHz) of **5**.

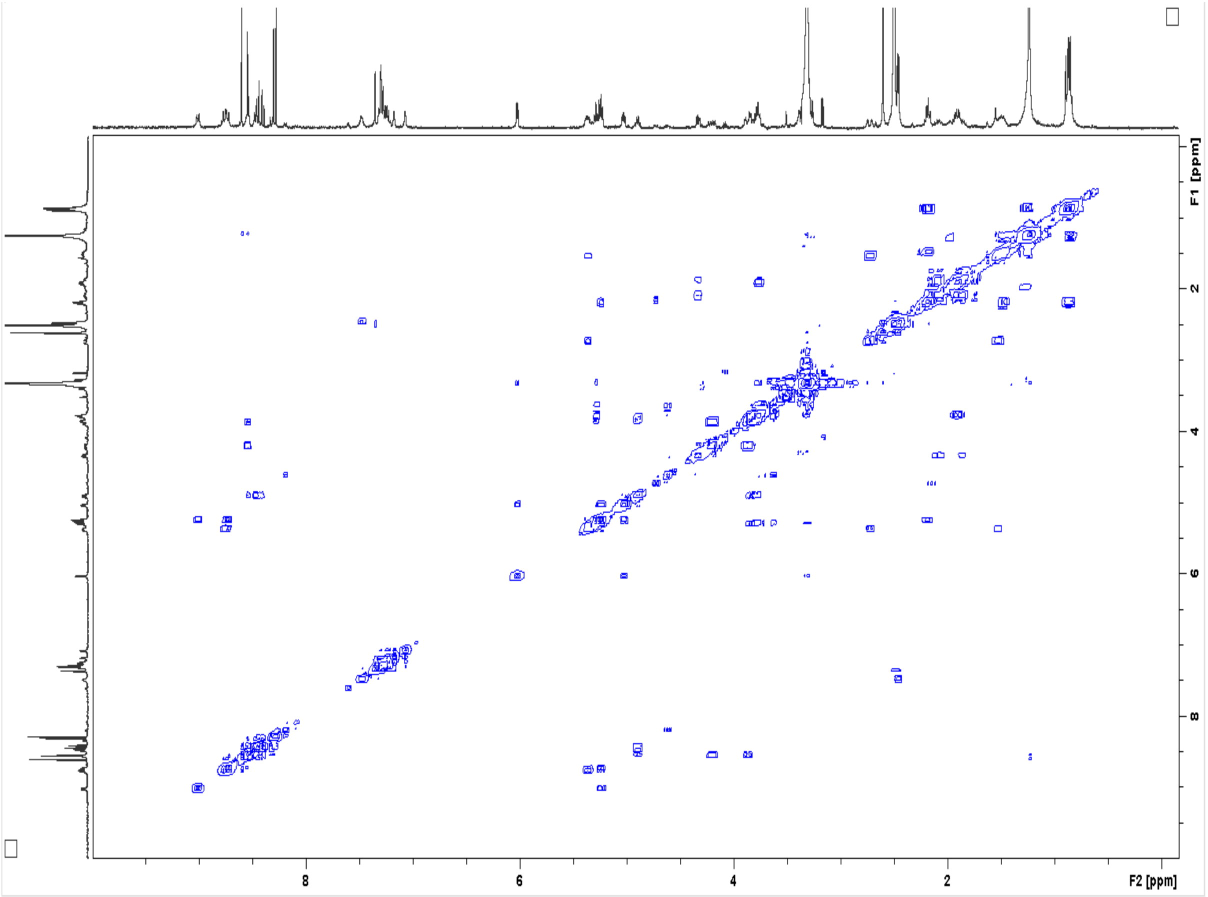

Figure S28. COSY spectrum of **5**.

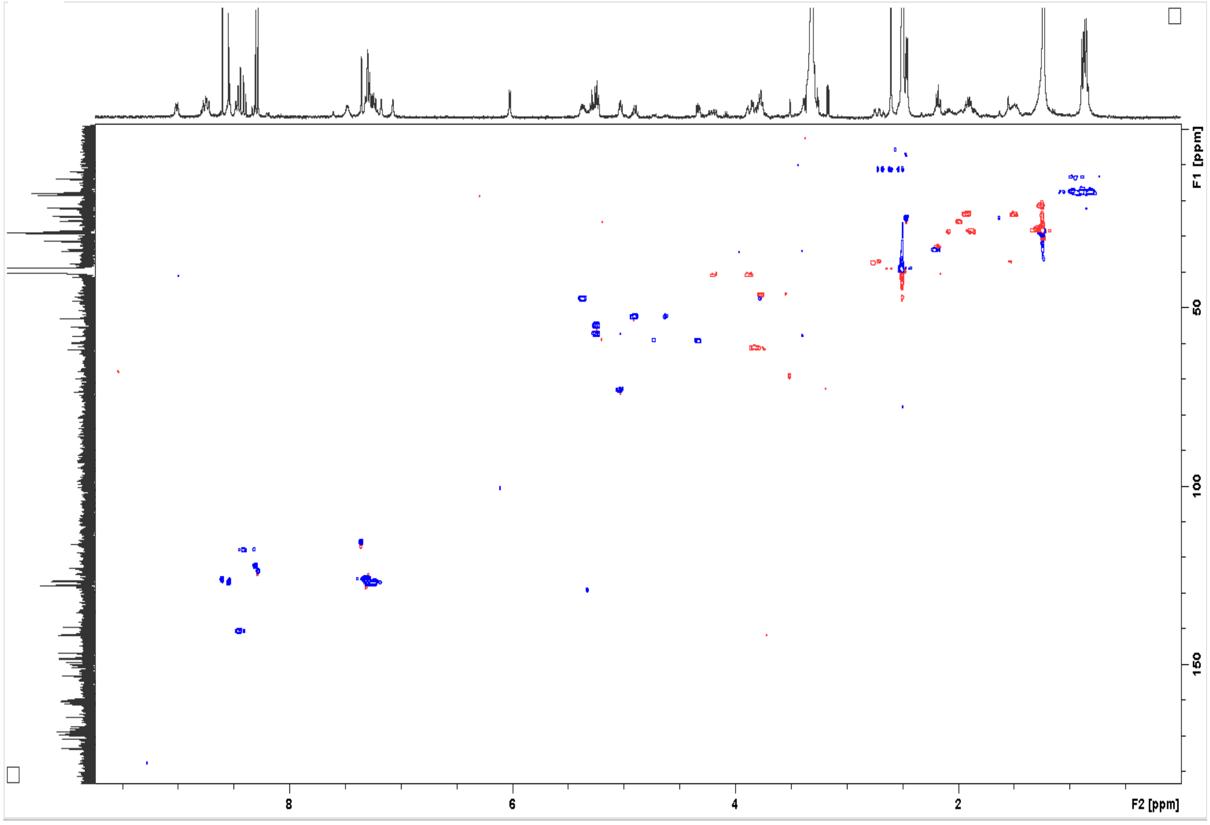

Figure S29. HSQC spectrum of **5**.

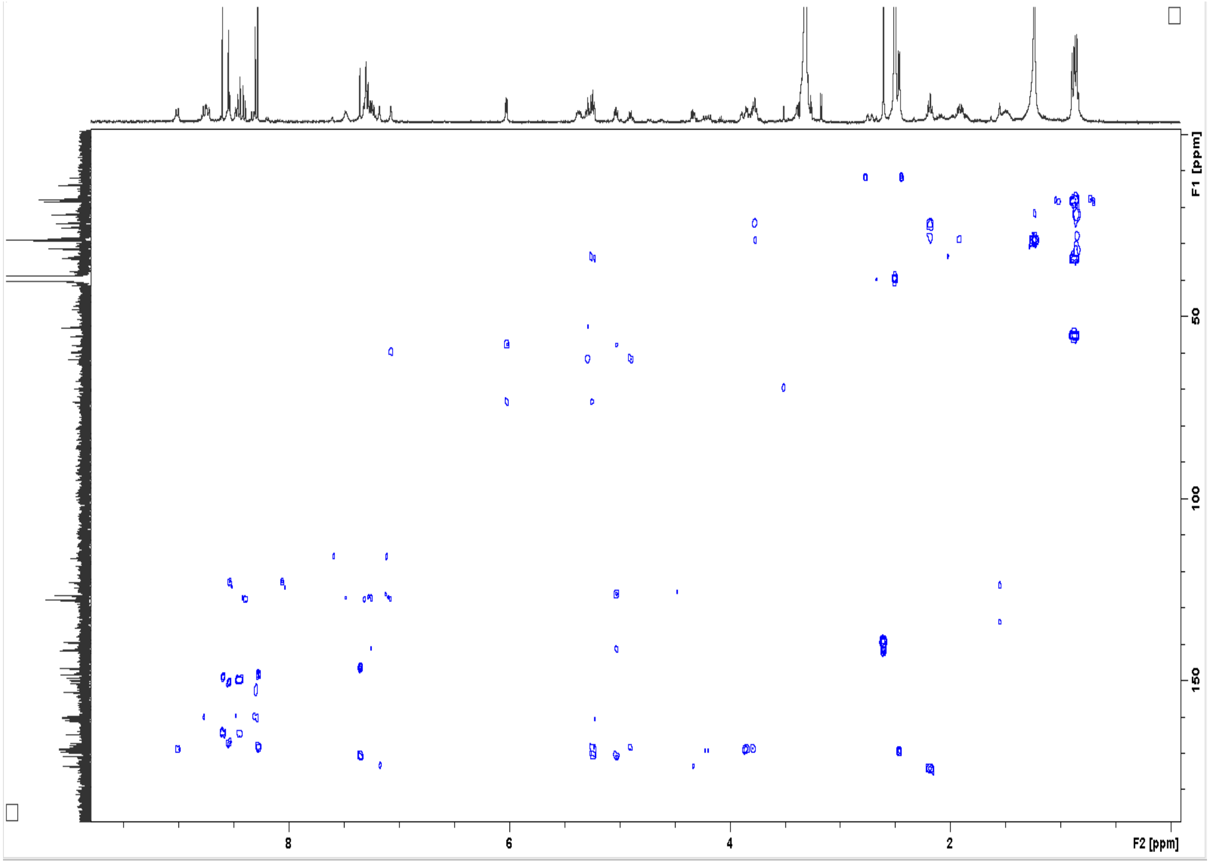

Figure S30. HMBC spectrum of **5**.

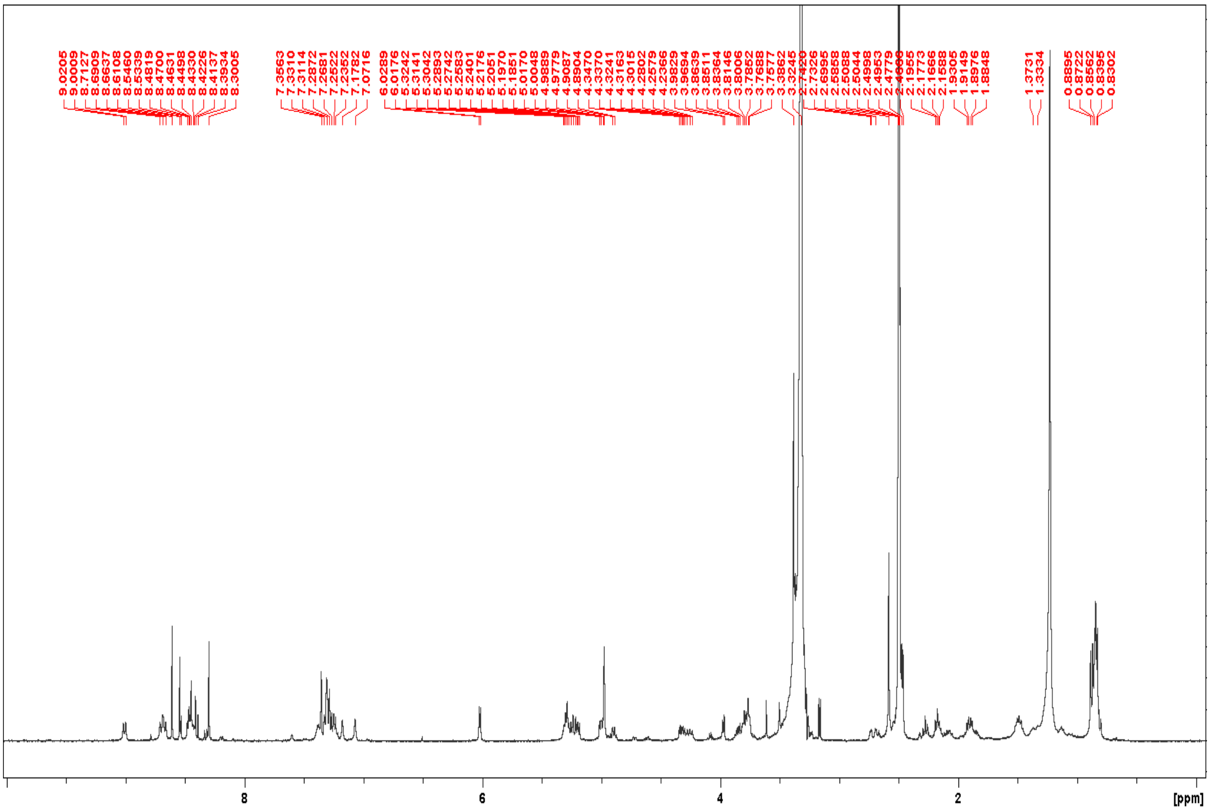

Figure S31. ^{1}H NMR spectrum (DMSO-d_6, 400 MHz) of **6**.

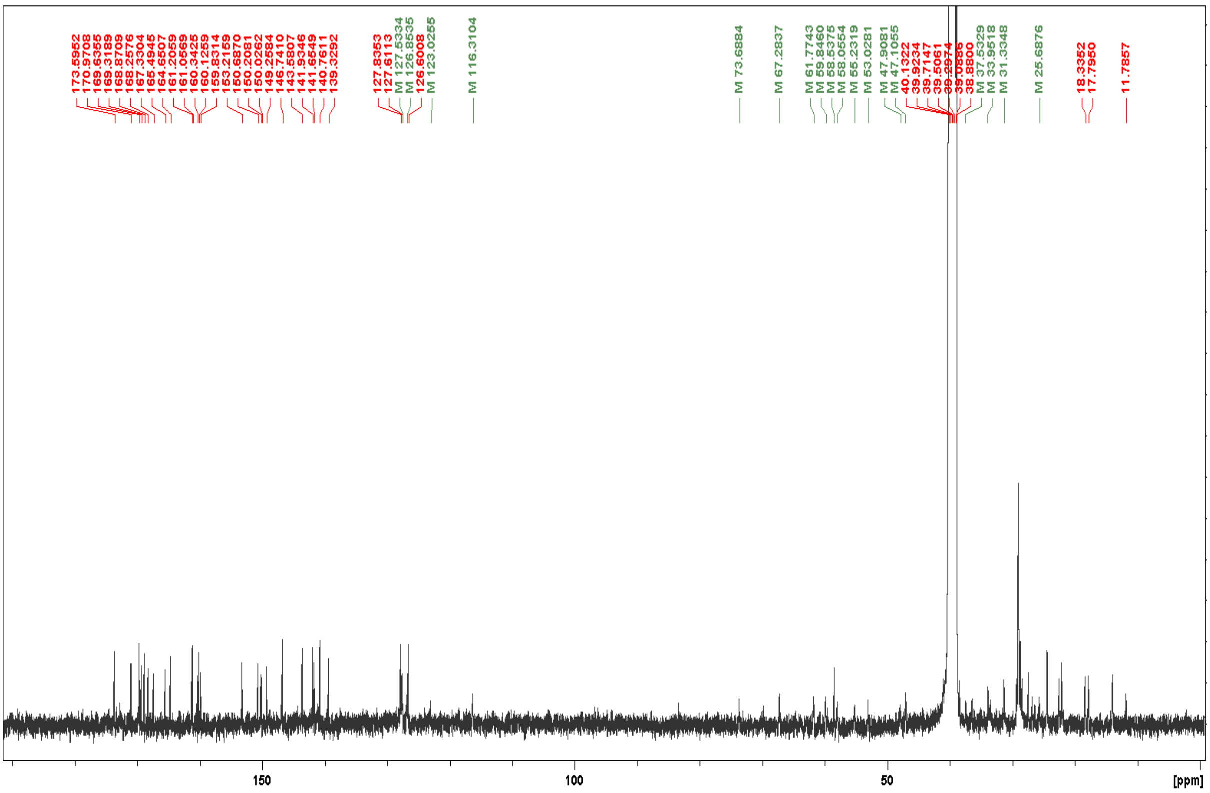

Figure S32. ^{13}C NMR spectrum (DMSO-d_6, 100 MHz) of **6**.

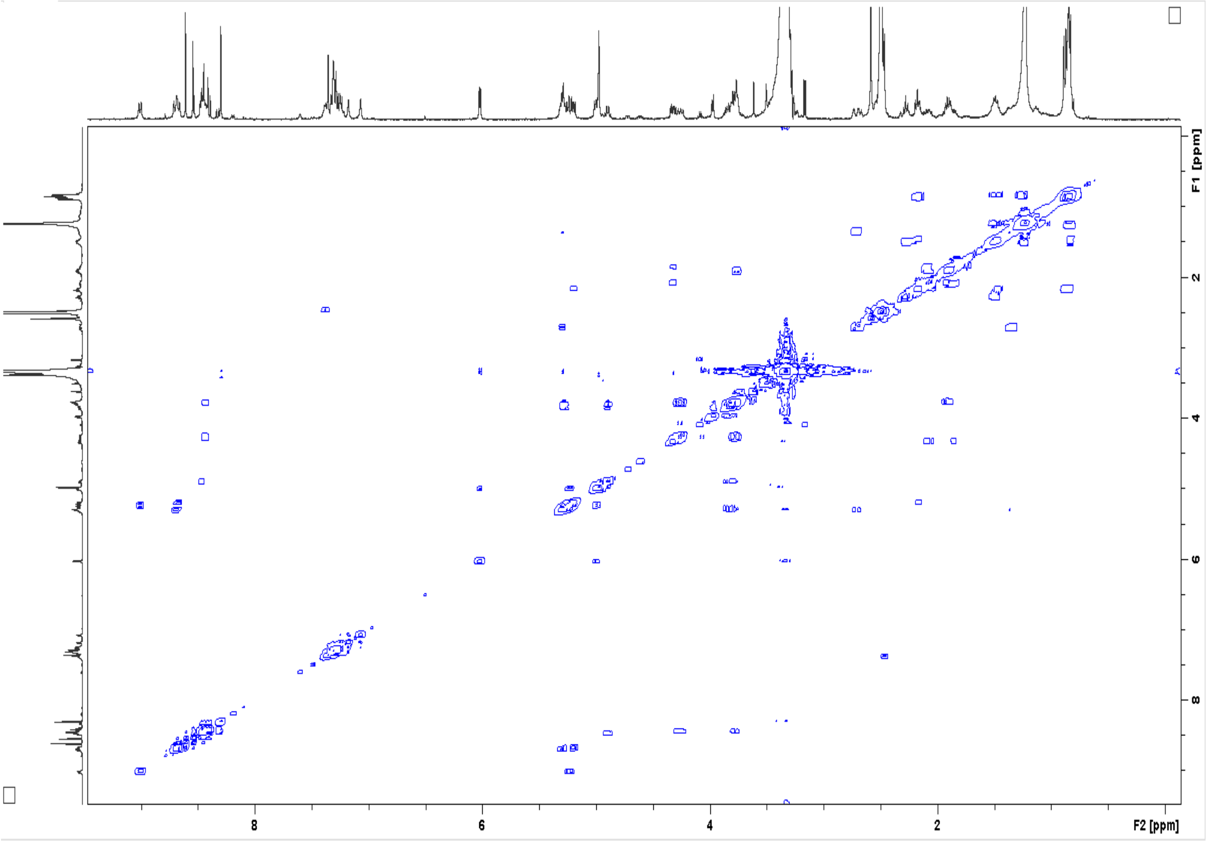

Figure S33. COSY spectrum of **6**.

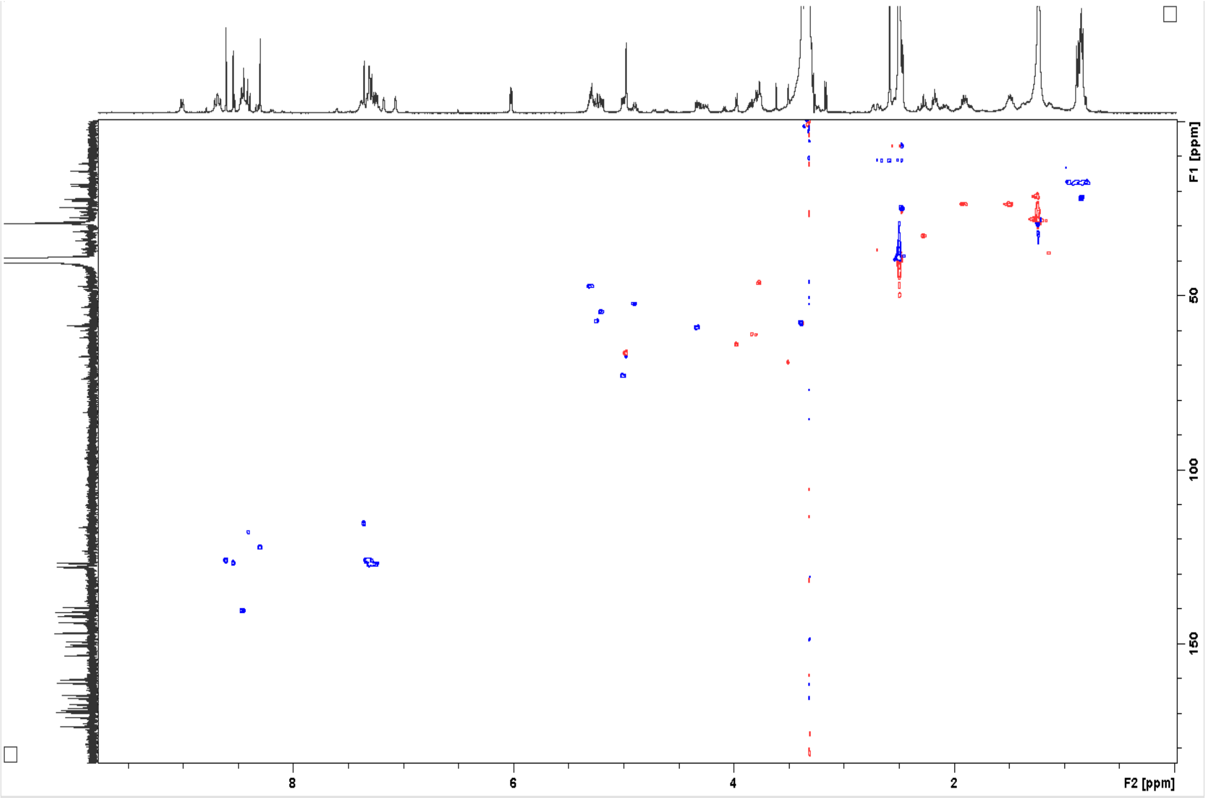

Figure S34. HSQC spectrum of **6**.

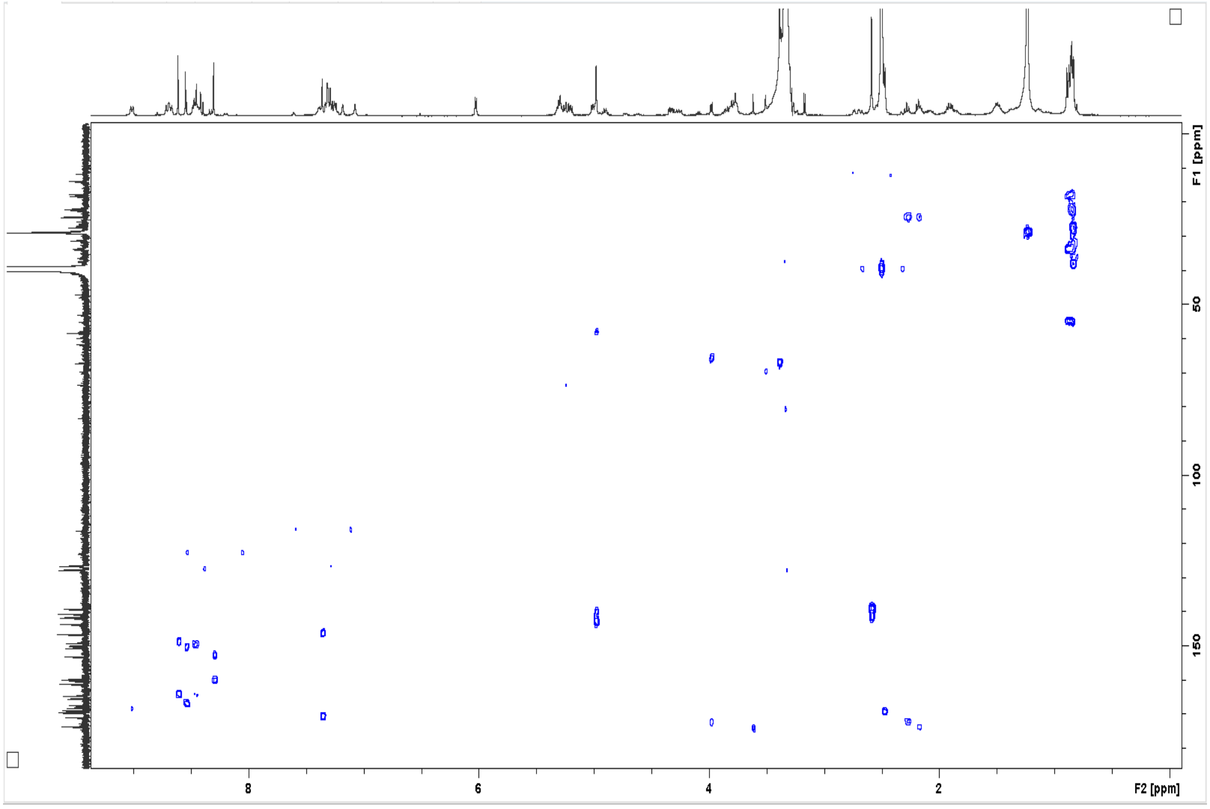

Figure S35. HMBC spectrum of **6**.

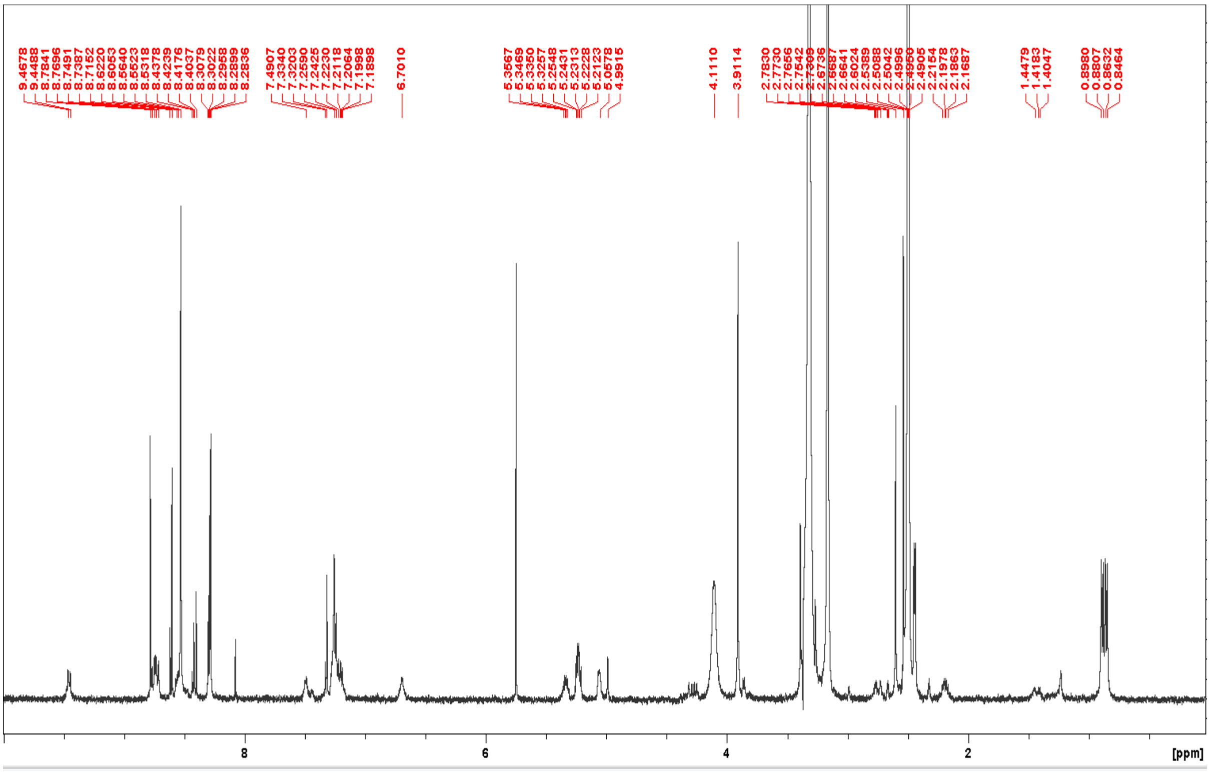

Figure S36. ^{1}H NMR spectrum (DMSO-d_6, 400 MHz) of **7**.

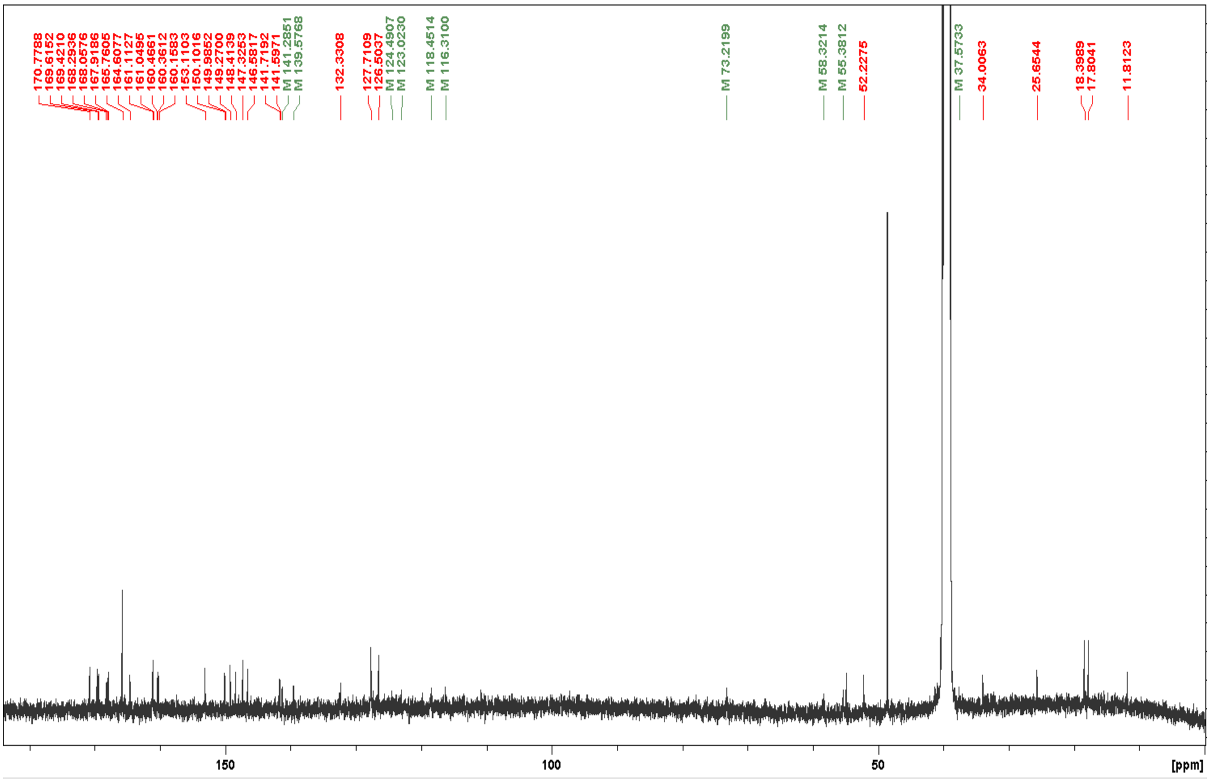

Figure S37. ^{13}C NMR spectrum (DMSO-d_6, 100 MHz) of **7**.

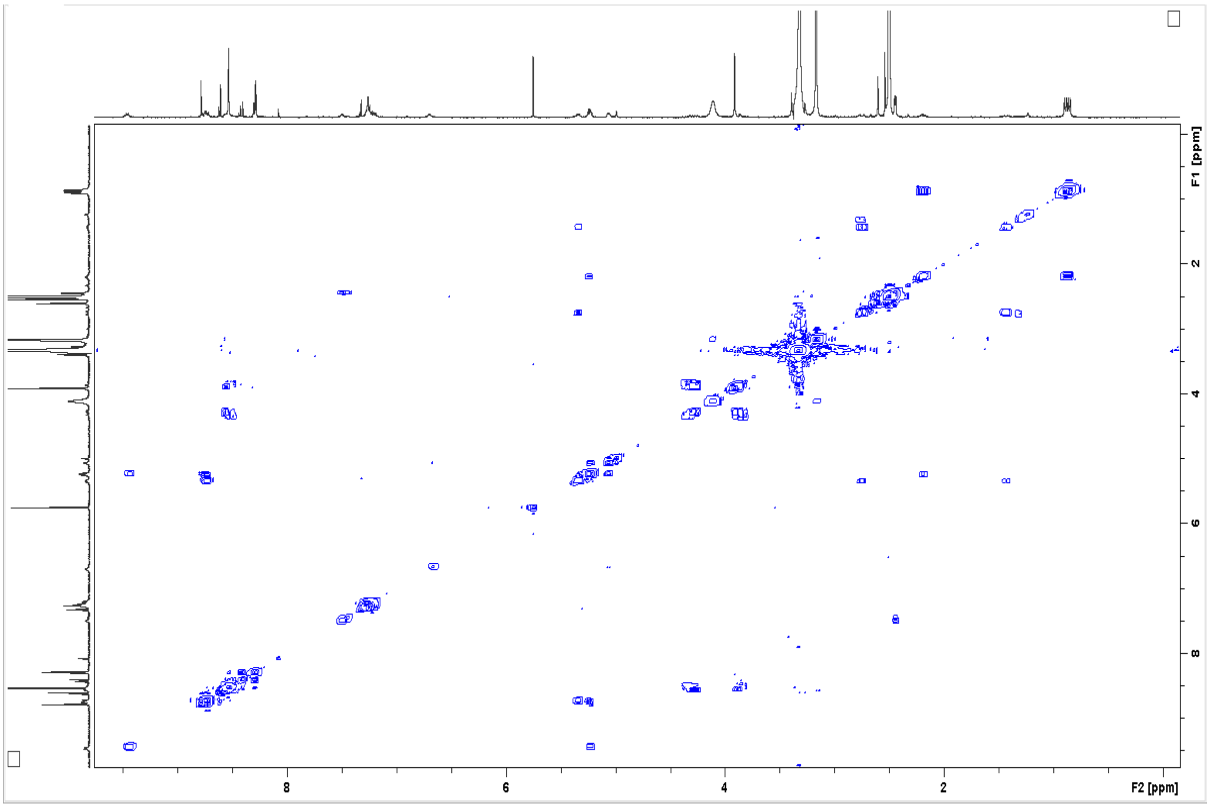

Figure S38. COSY spectrum of **7**.

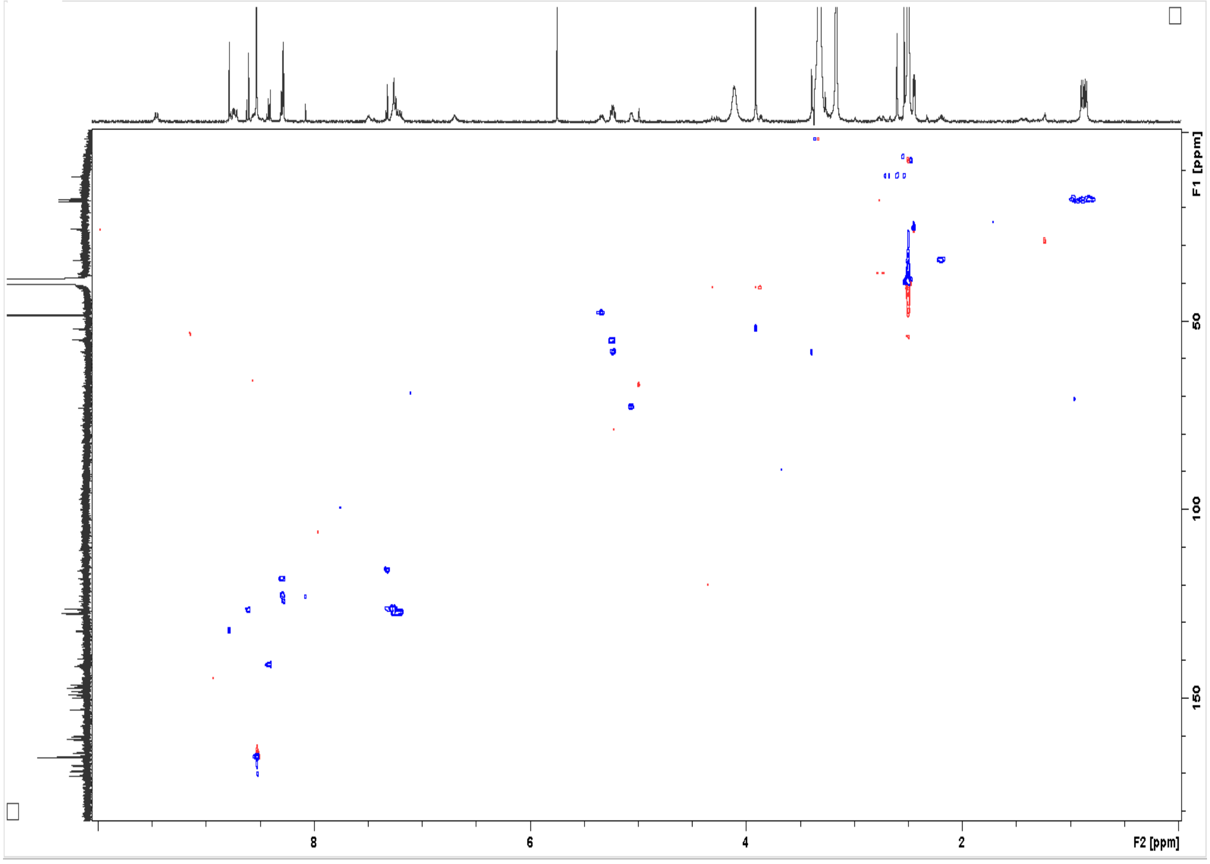

Figure S39. HSQC spectrum of **7**.

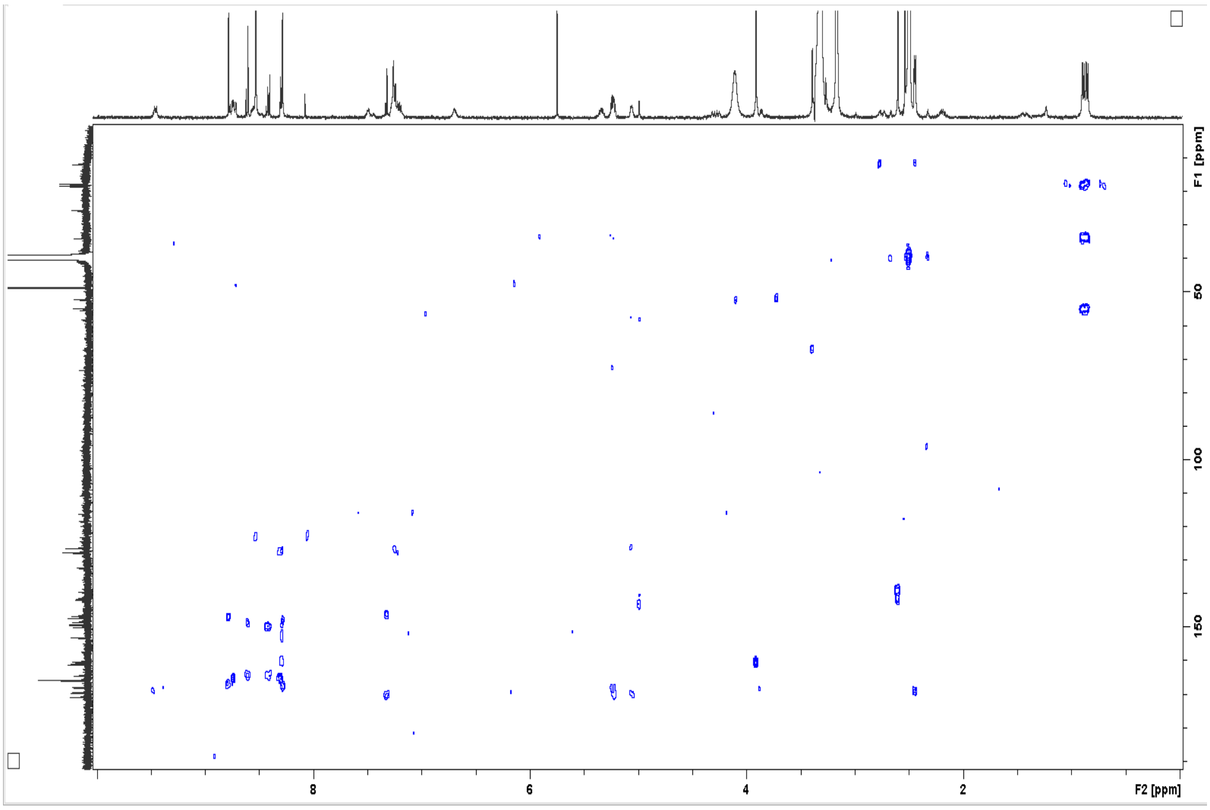

Figure S40. HMBC spectrum of **7**.

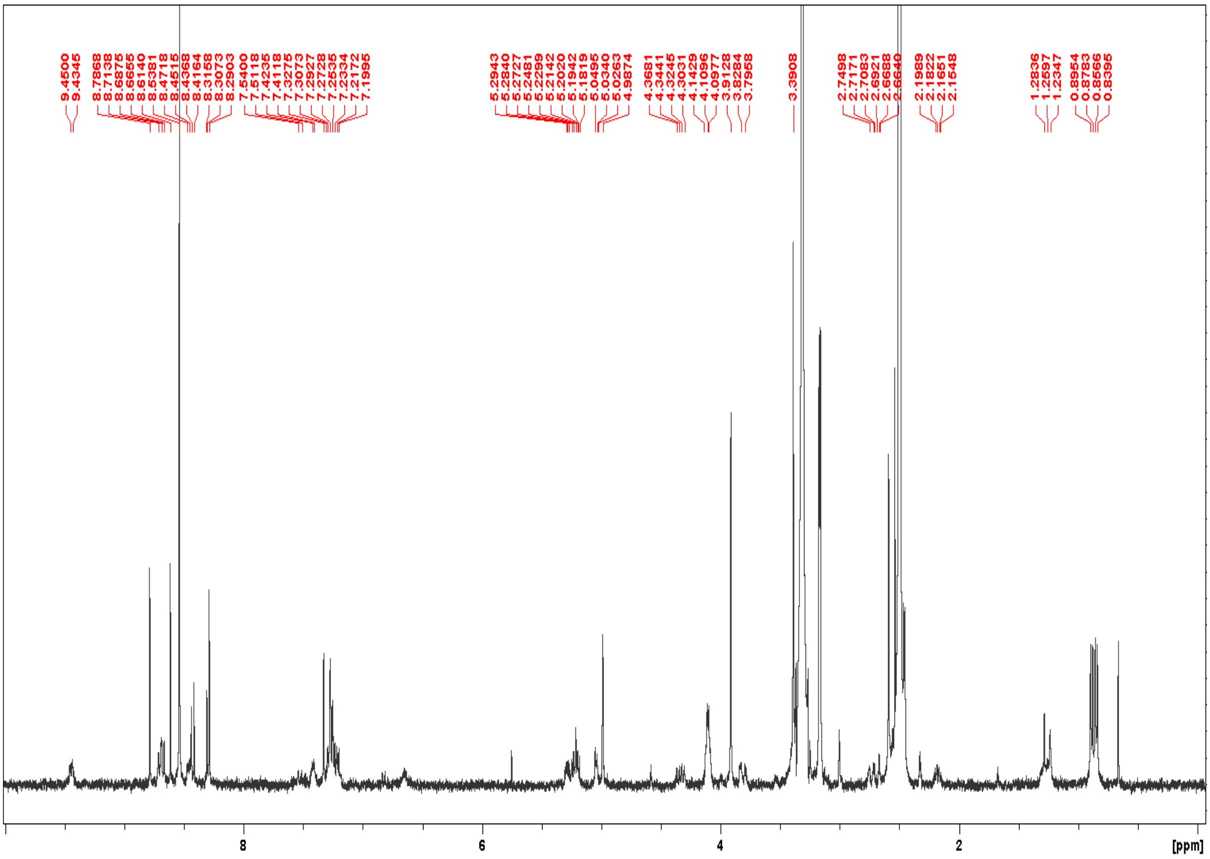

Figure S41. ^{1}H NMR spectrum (DMSO-d_6, 400 MHz) of **8**.

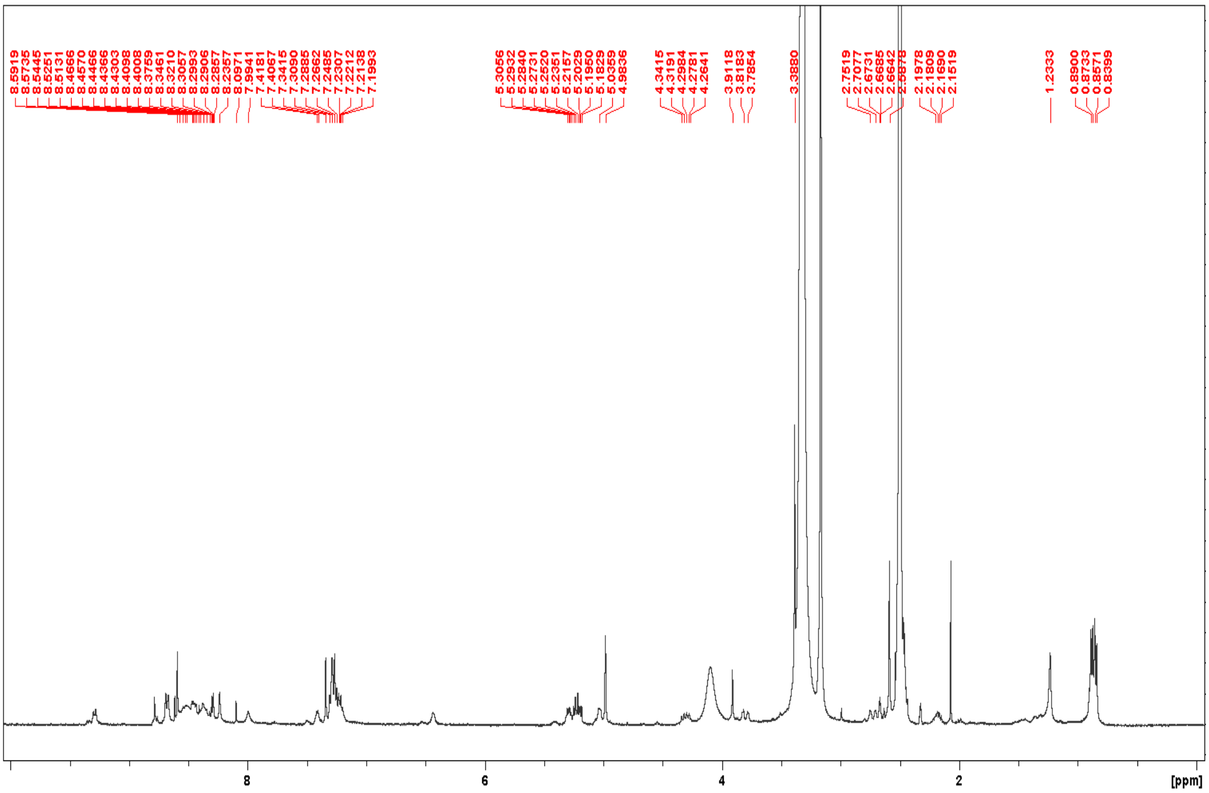

Figure S42. ^{1}H NMR spectrum (DMSO-d_6, 400 MHz) of **9**.

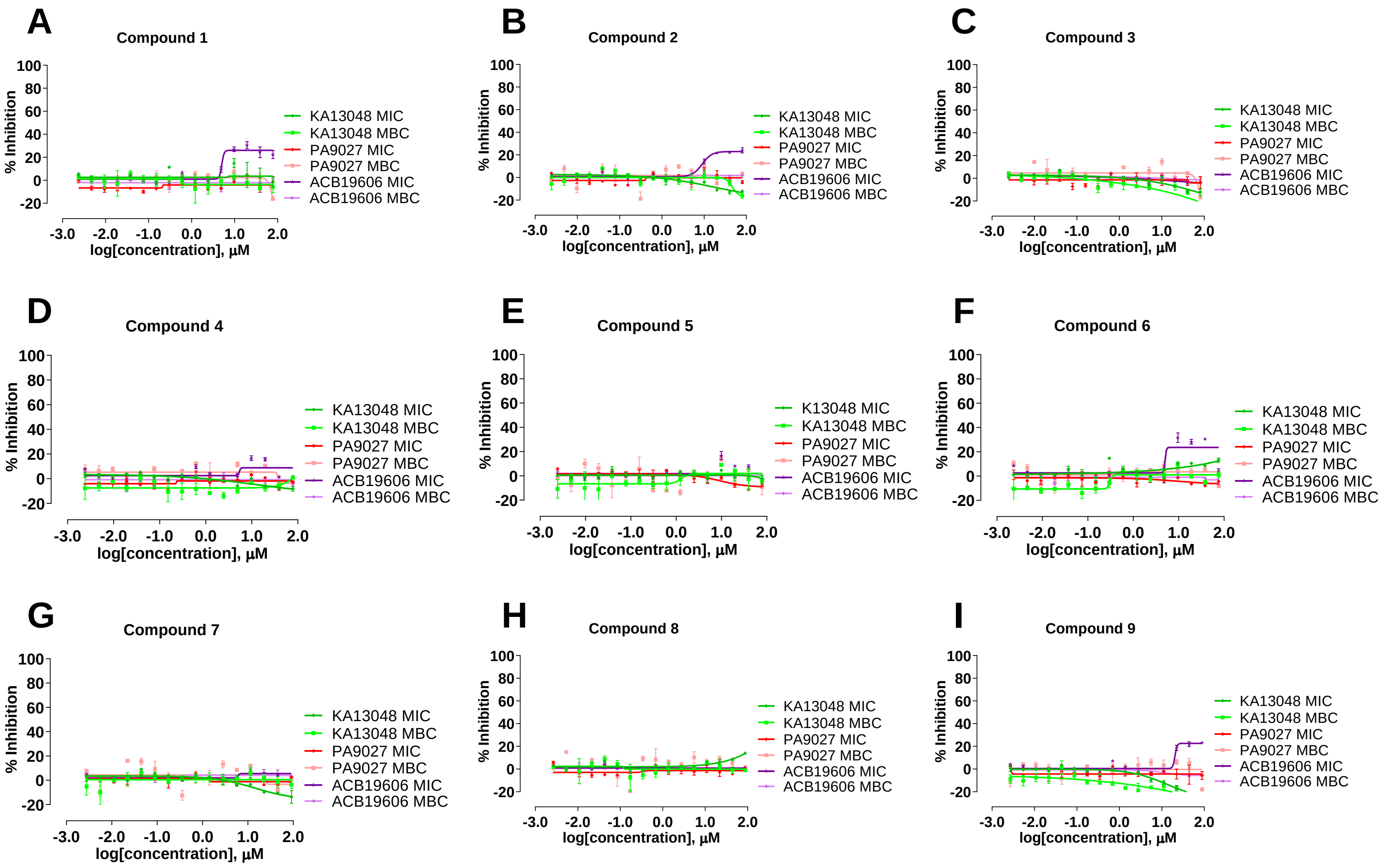

Figure S43. Inhibitory effect dose response curve against *Klebsiella aerogenes* (ATCC® 13048™), *Pseudomonas aeruginosa* (ATCC® 9027™) and *Acinetobacter baumannii* (ATCC® 19606™). **A)** Compound **1**, **B)** Compound **2**, **C)** Compound **3**, **D)** Compound **4**, **E)** Compound **5**, **F)** Compound **6**, **G)** Compound **7**, **H)** Compound **8**, **I)** Compound **9**.

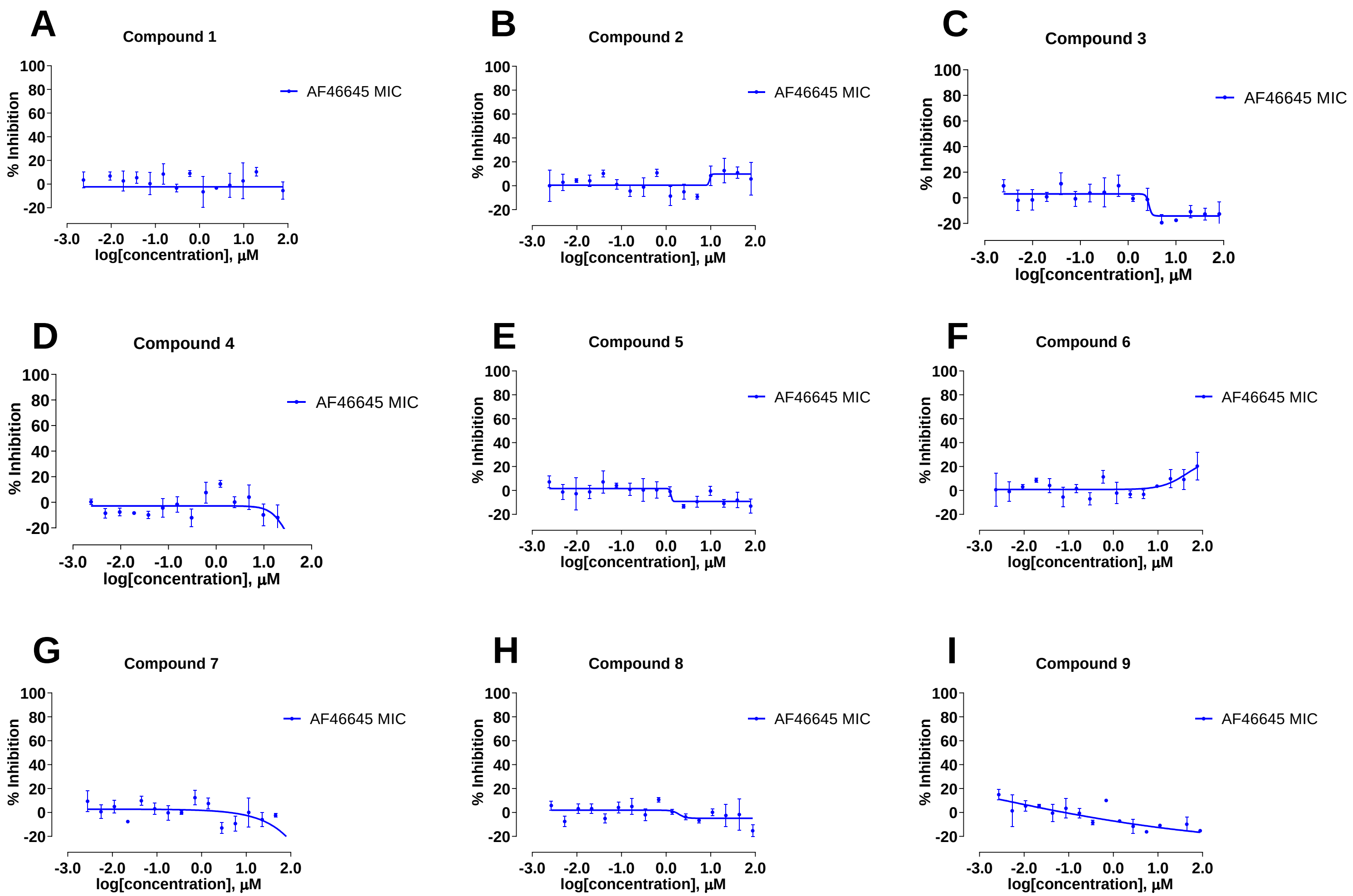

Figure S44. Inhibitory effect dose response curve against *Aspergillus fumigatus* (ATCC® 46645™). **A)** Compound **1**, **B)** Compound **2**, **C)** Compound **3**, **D)** Compound **4**, **E)** Compound **5**, **F)** Compound **6**, **G)** Compound **7**, **H)** Compound **8**, **I)** Compound **9**.

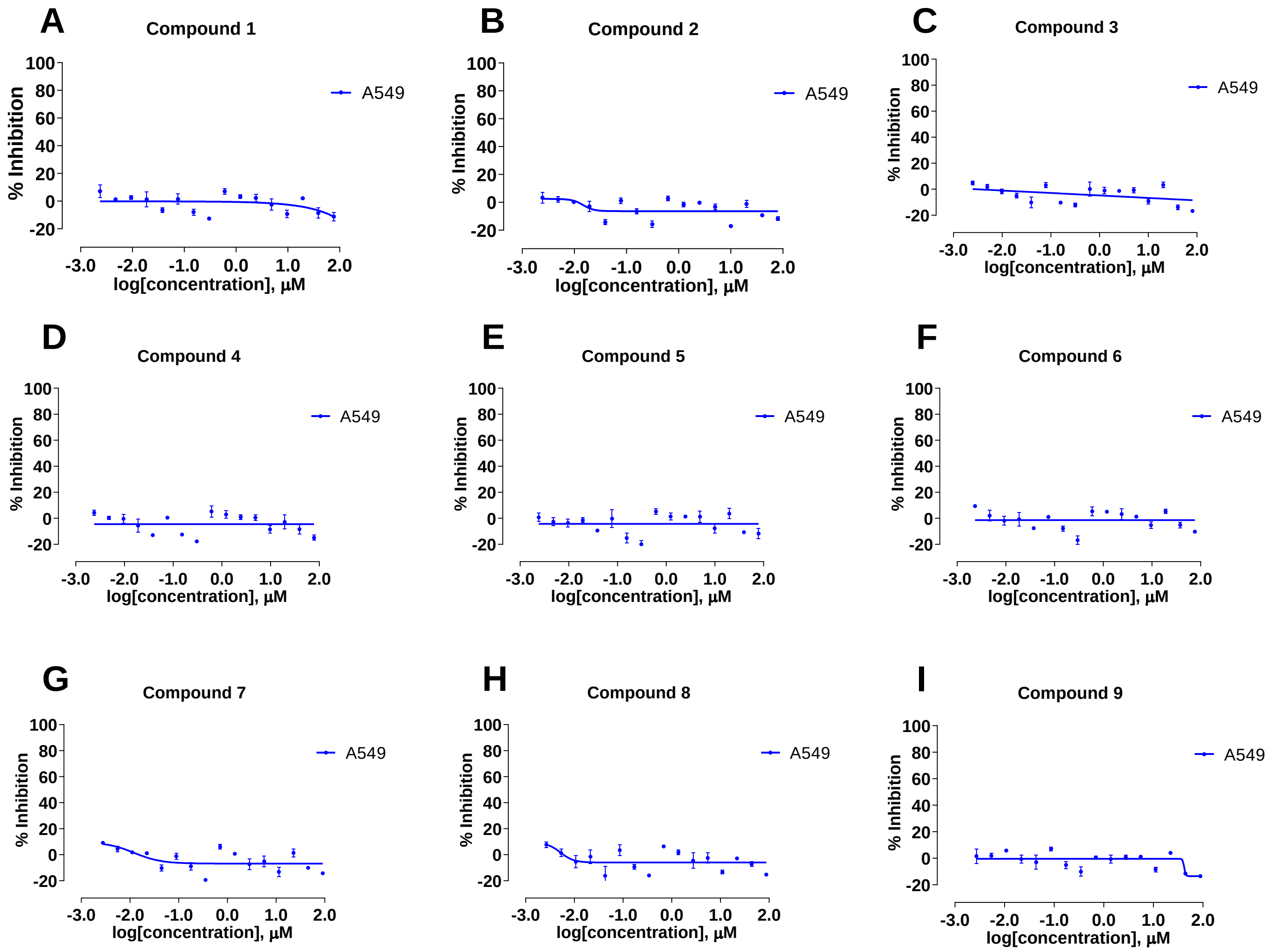

Figure S45. Inhibitory effect dose response curve against A549 Human lung carcinoma cells (ATCC® CCL-185™). **A)** Compound **1**, **B)** Compound **2**, **C)** Compound **3**, **D)** Compound **4**, **E)** Compound **5**, **F)** Compound **6**, **G)** Compound **7**, **H)** Compound **8**, **I)** Compound **9**.
